# Multimodal single-cell analyses of peripheral blood mononuclear cells of COVID-19 patients in Japan

**DOI:** 10.1038/s41598-023-28696-9

**Published:** 2023-02-02

**Authors:** Yukie Kashima, Taketoshi Mizutani, Kaori Nakayama-Hosoya, Saya Moriyama, Takayuki Matsumura, Yoshihiro Yoshimura, Hiroaki Sasaki, Hiroshi Horiuchi, Nobuyuki Miyata, Kazuhito Miyazaki, Natsuo Tachikawa, Yoshimasa Takahashi, Tadaki Suzuki, Sumio Sugano, Tetsuro Matano, Ai Kawana-Tachikawa, Yutaka Suzuki

**Affiliations:** 1 Laboratory of Functional Genomics, Department of Computational Biology and Medical Sciences, Graduate School of Frontier Sciences, The University of Tokyo, 5-1-5, Kashiwanoha, Kashiwa, Chiba 277-8562 Japan; 2 AIDS Research Center, National Institute of Infectious Diseases, Tokyo, Japan; 3 Research Center for Drug and Vaccine Development, National Institute of Infectious Diseases, Tokyo, Japan; 4 Department of Infectious Diseases, Yokohama Municipal Citizens’ Hospital, Kanagawa, Japan; 5 Department of Pathology, National Institute of Infectious Diseases, Tokyo, Japan; 6 Institute of Kashiwa-No-Ha Omics Gate, Kashiwa, Chiba Japan; 7 Joint Research Center for Human Retrovirus Infection, Kumamoto University, Kumamoto, Japan; 8 Department of AIDS Vaccine Development, Institute of Medical Science, University of Tokyo, Tokyo, Japan

**Keywords:** Viral infection, Infection, Next-generation sequencing

## Abstract

SARS-CoV-2 continues to spread worldwide. Patients with COVID-19 show distinct clinical symptoms. Although many studies have reported various causes for the diversity of symptoms, the underlying mechanisms are not fully understood. Peripheral blood mononuclear cells from COVID-19 patients were collected longitudinally, and single-cell transcriptome and T cell receptor repertoire analysis was performed. Comparison of molecular features and patients’ clinical information revealed that the proportions of cells present, and gene expression profiles differed significantly between mild and severe cases; although even among severe cases, substantial differences were observed among the patients. In one severely-infected elderly patient, an effective antibody response seemed to have failed, which may have caused prolonged viral clearance. Naïve T cell depletion, low T cell receptor repertoire diversity, and aberrant hyperactivation of most immune cell subsets were observed during the acute phase in this patient. Through this study, we provided a better understanding of the diversity of immune landscapes and responses. The information obtained from this study can help medical professionals develop personalized optimal clinical treatment strategies for COVID-19.

## Introduction

Severe acute respiratory syndrome coronavirus 2 (SARS-CoV-2), responsible for coronavirus disease 2019 (COVID-19), continues to spread worldwide. Since the start of the SARS-CoV-2 pandemic, diverse clinical symptoms of COVID-19 have been reported, which are substantially distinct between different patients, and have therefore been the focus of scientific and clinical interest. While some patients experience severe symptoms, others do not. In many cases, a SARS-CoV-2 infection results in only asymptomatic infections, which can cause the spread of the infection without the infected individuals being aware of it. Intensive epidemiological studies have revealed that aging is the most important factor affecting the risk of severe COVID-19^1^. According to the COVID-19 data portal in the United States Centers for Disease Control and Prevention, the number of hospitalizations is 80 times higher and the number of deaths 7900 times higher in older people^2–5^. It is also suggested that different ethnicities respond differently to COVID-19, with Asian and non-Hispanic people at a lower risk of infection, hospitalization, and death^6^. However, which cell populations play a role in the diverse symptoms of COVID-19 remains largely unknown. The present data on multimodal and longitudinal representation of the cells and their diversity in populations are still limited to further understand the molecular etiology and epidemiology of COVID-19.

From a molecular viewpoint, various immune responses have been associated with COVID-19 symptoms. In particular, the T cell pathway plays a pivotal role in various molecular systems associated with these immune responses. T cell receptor (TCR) diversity appears to be one of the most important factors in determining the effect of COVID-19 on infected individuals^7^. It is supposed that a rich TCR diversity may play a role in inducing more effective immune responses^7^. It has also been shown that the diversity of TCR repertoires declines in elderly people, thereby increasing the chance of diseases becoming more severe^8^. Previous studies have shown that SARS-CoV-2-specific T cells are detected during the convalescent period^9,10^. Additionally, some studies have reported pre-existing cross-reactive SARS-CoV-2 T cells in unexposed individuals^11,12^. These cross-reactive T cells, previously induced by other human coronaviruses (HCoVs), are believed to elicit T-cell responses against SARS-CoV-2 immediately after infection^13^. Although there are many studies on the TCR repertoire using convalescent and unexposed donor samples, TCR analysis in the early infection stage is limited by sample availability.

In this study, we attempted single-cell RNA sequencing analysis (scRNA-seq) of peripheral blood mononuclear cells (PBMC). We also conducted single-cell T-cell receptor (TCR) sequencing (scVDJ-seq) to monitor the T cell immune status. Eight participants were selected: two healthy control donors, and six COVID-19 patients of varying ages and clinical symptoms. Samples were collected from patients before spring 2020, when only the original strain was found in Japan. None of the patients were vaccinated. The samples were collected in the early days of infection in order to record the responses of early-stage infection stage.

## Results

### Single cell RNA sequencing analysis of PBMCs from COVID-19 patients

**Figure 1:**
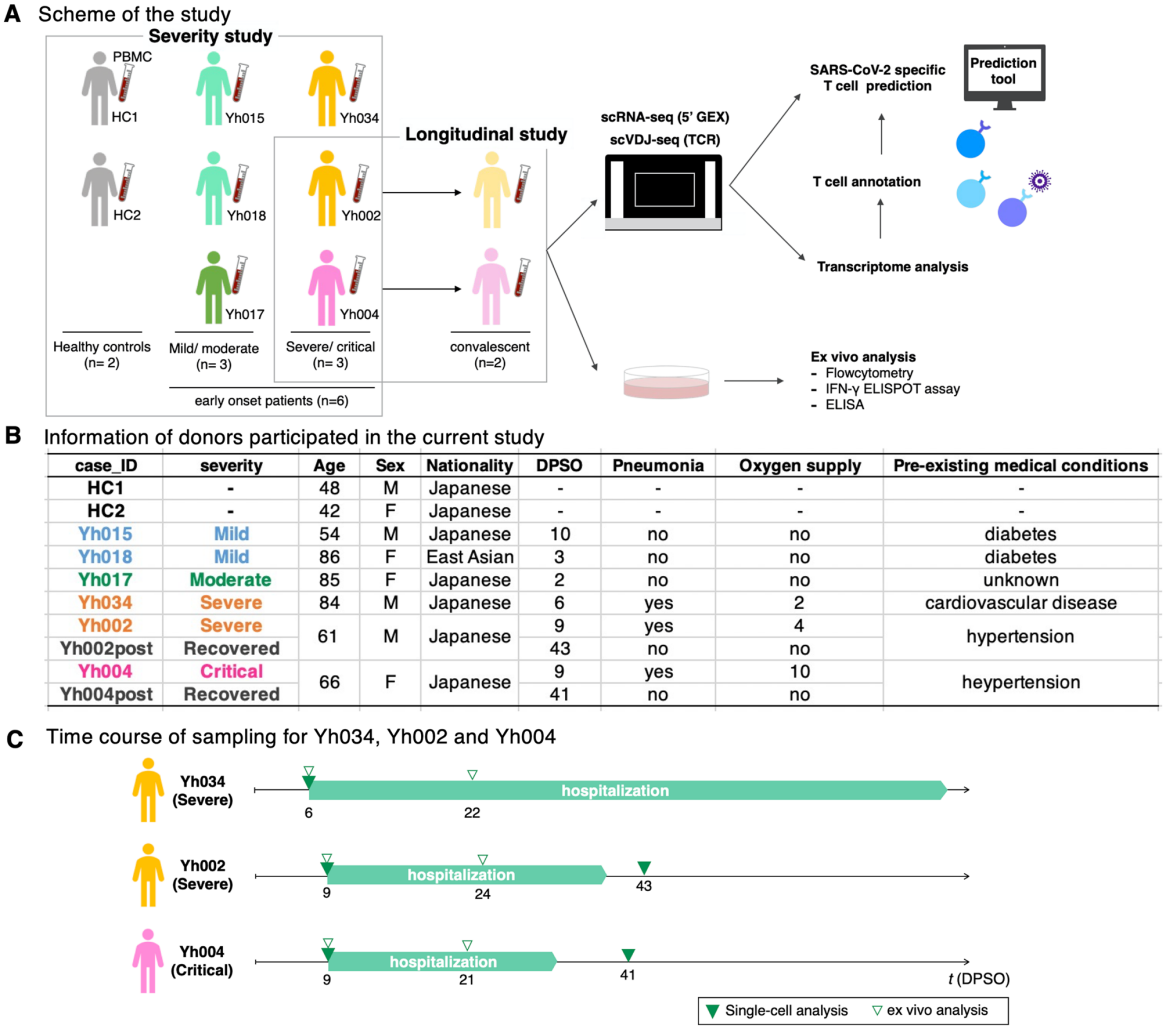
Strategy of the current study.** (A)** PBMCs are separated from whole blood samples and analyzed by 10 × Genomics Chromium scRNA-seq and scVDJ-seq (TCR). Our datasets include two healthy donors and six COVID-19 patients: two mild, a moderate, two severe, and a critical patient. Those patients are divided into two groups: mild/moderate (Yh015, Yh018, and Yh017) and severe/critical (Yh034, Yh002, and Yh004). **(B)** Inset table shows donors’ basic and clinical information. DPSO shows the timepoint of days post symptom onset at single-cell analysis. **(C)** Time course of sampling for Yh002, Yh004, and Yh034. The period of hospitalization and timepoint of analysis are shown. Legend is shown in the margin.

To study the transcriptome profiles of PBMCs in COVID-19 patients, blood samples were collected from six patients: two mild (Yh015 and Yh018), one moderate (Yh017), two severe (Yh034 and Yh002), and one critical (Yh004) (Fig. 1A–C). RT-PCRs confirmed SARS-CoV-2 infections in all patients. PBMCs from the blood samples on the day of or the day after hospital admission, i.e., 2–10 days post symptom onset (DPSO), were used for single-cell RNA sequencing (scRNA-seq) to analyze transcriptome profiles at an early infection stage. No treatment intervention was initiated at the time of sampling.

scRNA-seq was conducted using the 10 × Genomics Chromium system. PBMCs from two healthy donors were also analyzed. The statistics for the generated sequence data are presented in Supplementary Table S1.

### Analysis of representative cellular populations during acute infection

**Figure 2:**
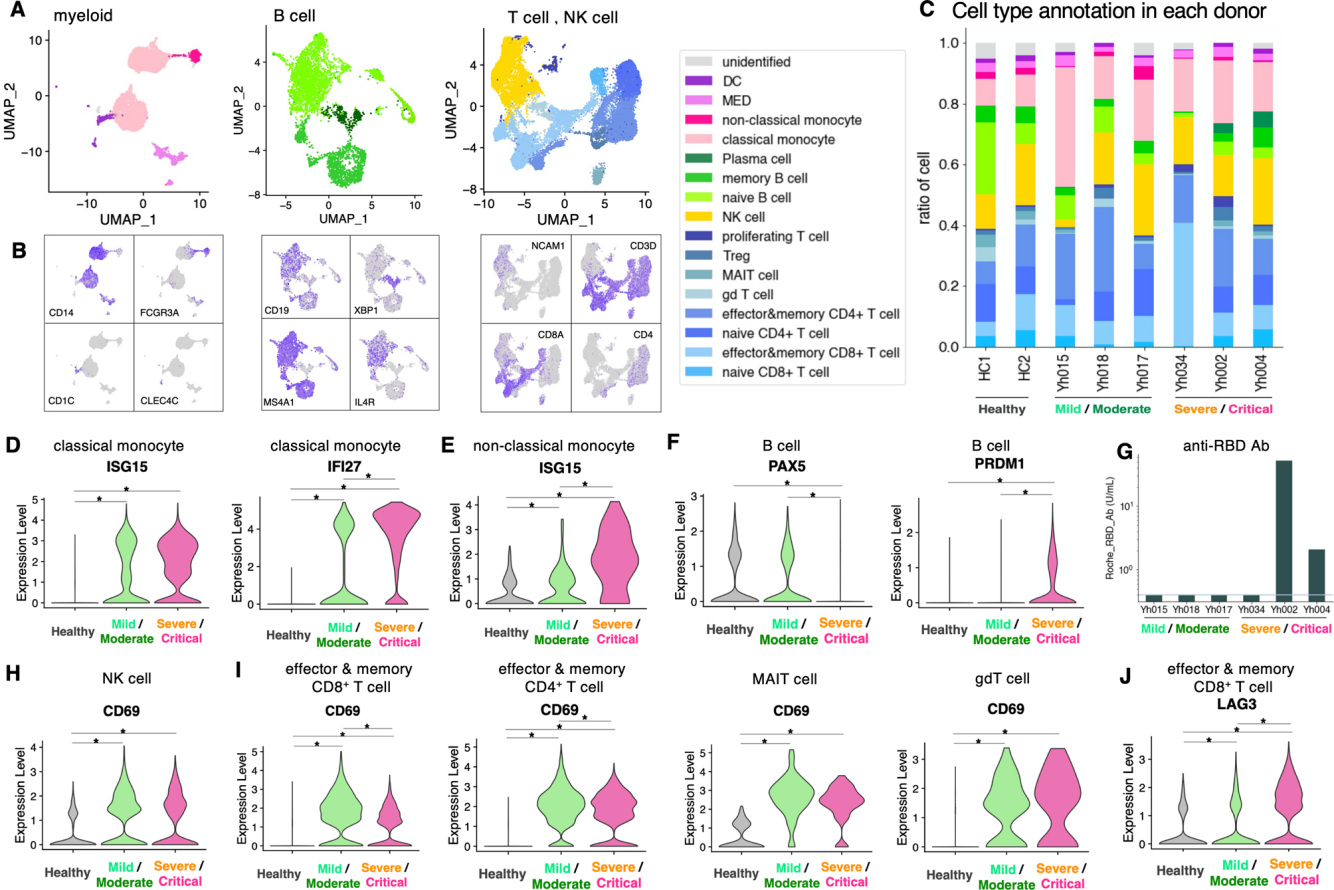
Severity-dependent character in COVID-19 patients. **(A)** Annotation of cells used in the severity dependent study of myeloid (classical monocyte, non-classical monocyte, MED, and DC), B cell (naïve B cell, memory B cell, and Plasma cell), and T cell and NK cell (naïve CD8^+^ T cell, effector & memory CD8^+^ T cell, CD4^+^ naïve T cell, CD4^+^ effector & memory T cell, gd T cell, MAIT cell, Treg, proliferating T cell and NK cell) shown in UMAP. Color legends are shown in the margin. **(B)** Feature plots of representative markers for myeloid, B cell, T cell, and NK cell. Detailed information of annotation is shown in Supplementary Figs. S1, S2, S3 and the methods. **(C)** Proportion of cells present in each case. Bar plot shows cell proportions in healthy donors and COVID-19 patients. Color legends the same as **(A)**. **(D, E)** Expression level of inflammatory genes in classical monocytes **(D)** and non-classical monocytes **(E)**. **(F)** Expression levels of PAX5 and PRDM1 in B cells. **(G)** SARS-CoV-2 anti-RBD antibody levels in patients. The *x-axis* shows the patient, and the *y-axis* shows antibody levels (U/ mL). Red line shows the limit of detection (0.4). **(H, I)** Expression of activation marker CD69 in NK cells **(H)**, and effector & memory CD8^+^ T cells, effector & memory CD4^+^ T cells, MAIT cells and gd T cells **(I)**. **(J)** Expression of exhaustion marker LAG3 in effector & memory CD8^+^ T cells. In the violin plot (**D**–**F**) and (**H**-**J**), the healthy group includes cells from two control cases (HC1 and HC2), mild/moderate from three cases (Yh015, Yh018, and Yh017), and severe/critical from three cases (Yh034, Yh002, and Yh004). Statistically significant differences are marked with * (p < 0.05, Wilcoxon Rank Sum test).

First, the cell types were annotated using known marker genes (Fig. 2A, B, and Supplementary Figs. S1–S6). In the following section, we inspected the following representative cell populations: (i) Myeloid cells, (ii) B cells, (iii) NK cells, and (iv) T cells. The overall proportion of cells present, and their gene expression patterns were examined in the patient groups with distinct clinical symptoms.

#### Myeloids

The myeloid fraction showed the most drastic changes in COVID-19 patients^14^. Consistent with a previous report^14^, classical monocytes were the most induced fraction in SARS-CoV-2 infections, with similar results in both mild and severe patients (Fig. 2C and Supplementary Fig S7A). As some of the COVID-19 patients in the current study is over 70 y.o. (Fig. 1B), cell ratios were compared to age-controlled healthy individual^15^ and demonstrated classical monocyte induction (Supplemental Fig. S8). Gene expression levels were then analyzed. Inflammatory-related genes, such as ISG15 and IFI27, were upregulated in COVID-19 patients (Fig. 2D). In contrast, the non-classical monocyte population tended to decrease in severe/critical cases (Fig. 2C and Supplementary Fig. S7A). Upregulation of the inflammatory-related gene ISG15 was also observed in non-classic monocytes, especially in severe/critical cases (Fig. 2E).

#### B cells

The population size of naïve B cells was smaller in severe cases than in mild cases (Fig. 2C and Supplementary Fig. S7B). In contrast, a relative abundance of plasma cells was observed in severe cases, especially in Yh002 and Yh004 (Fig. 2C). The gene expression levels of the representative markers of B cell development (low and high expression of PAX5 and BLIMP-1, respectively) also clearly revealed the abundance of terminally differentiated plasma cells in severe cases (Fig. 2F). In fact, the receptor-binding domain (RBD)-specific antibody was only detected in Yh002 and Yh004, in which a relatively high frequency of plasma cells was also observed (Fig. 2G), suggesting that SARS-CoV-2-specific B cells were stimulated and differentiated into antibody-producing plasma cells within two weeks after infection in the two severe cases. This result is further supported by a previous report showing the correlation between antibody-secreting cells and the severity of COVID-19^16^.

#### NK cells

Previous studies have shown a reduced but definite activation of NK cells during the early phase of COVID-19^17,18^. In this study, the total size of the NK cell population did not show a substantial change in the early days of SARS-CoV-2 infection (Fig. 2C and Supplementary Fig. S7C). However, close examination of the gene expression in NK cells revealed that NK cells were in an active state in COVID-19 patients (Fig. 2H).

#### T cells

A lower number of naïve cells and higher number memory cells was observed in both CD4^+^ and CD8^+^ T cells in COVID-19 patients than in healthy controls (Fig. 2C and Supplementary Fig. S7D). As the naïve/memory ratio in T cells decreases with age, this may reflect the older age of COVID-19 patients. Although the relative percentages of other T cell populations were low, MKI67 (Ki67)-expressing proliferating T cells were more abundant in severe/critical patients compared to healthy controls and mild patients, while gamma delta T cell and MAIT cell populations were reduced in patients with acute COVID-19 (Fig. 2C and Supplementary Fig. S7E). CD69 expression showed increased upregulation in each T cell population (CD4^+^ and CD8^+^ effector and memory T, gdT, and MAIT) in acute COVID-19 patients compared to healthy controls (Fig. 2I), suggesting that T cells were activated under inflammatory conditions. Notably, not only the activation marker CD69 but also the exhaustion marker LAG3 were upregulated in CD8^+^ T cells in severe/ critical cases (Fig. 2J).

Taken together, although the frequency of each cell subset differed between individuals, these observed changes were generally consistent with previous reports^14,19–22^. The statistical significance of this difference, especially regarding cell proportion, was not confirmed in the present study because of the relatively small sample size.

### Effect of ethnicity on immune responses

Next, we examined the effect of ethnicity on COVID-19 severity. We compared our dataset to that of a previous study conducted in another country. We used data of patients of similar ages^14^ to those of the present study to compare the representative proportion of cells present (Supplementary Fig. S9). Similar patterns were observed for the mild/moderate patients. In severe/critical patients, our dataset showed a lower proportion of myeloid cells. The reduced myeloid response may be associated with the lower proportion of COVID-19 deaths in Japan, however due to the small difference and sample size, this should be further evaluated by a study looking at a larger cohort.

### TCR profiling and SARS-CoV-2-specific T cell responses during acute infection

To further determine the characteristics of T cells in COVID-19 patients, TCR sequencing was performed using the 10 × Genomics Chromium V(D)J system. The intermediate products of the corresponding scRNA-seq libraries were utilized for libraries construction so that the cell barcodes could be shared between the scVDJ-seq and scRNA-seq libraries (Fig. 1A).

**Figure 3:**
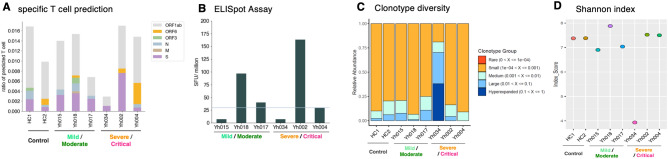
TCR diversity and SARS-CoV-2 specific T cell responses in COVID-19 patients.** (A)** Ratio of T cells predicted to be specific to SARS-CoV-2 in two healthy controls and six COVID-19 patients. HC1(healthy), HC2 (healthy), Yh015 (mild), Yh018 (mild), Yh017 (moderate), Yh034 (severe), Yh002 (severe), and Yh004 (critical) colored by recognized antigens. The *y-axis* shows the frequency of T cells predicted to SARS-CoV-2 specific by TCRex. Color key is shown in the margin. **(B)** SARS-CoV-2 specific T cell population analyzed ex vivo. For ELISpot assay, peptides corresponding to the whole SARS-CoV-2 antigen are used, excluding ORF1ab. Red line shows the threshold (30 SFC/million PBMCs). The results for the controls are shown in Supplementary Fig. S11. **(C, D)** TCR clonotype diversity of Yh034, Yh002, and Yh004.

The possible epitopes of SARS-CoV-2 for each TCR were predicted by the reference epitope database, TCRex^23^. An average of 1.2% of the annotated T cells were predicted to express TCR, which may recognize peptides derived from SARS-CoV-2 proteins (Fig. 3A and Supplementary Table S2). In all COVID-19 cases, the major predicted target region of T cell recognition was ORF1ab. However, it also predicted that 1.3% of T cells (1.6% for HC1, 1.0% for HC2) in healthy controls to recognize SARS-CoV-2 protein and most frequent target was ORF1ab (Fig. 3A and Supplementary Table S2). This prediction is based only on the TCR sequence, not on the function of the T cell. Therefore, the number of SARS-CoV-2-specific T cells was quantified using IFN-γ ELISpot assays and compared to the predicted number of SARS-CoV-2-specific TCRs in each patient (Fig. 3A, B). A mixture of overlapping peptides encoding the entire proteome of SARS-CoV-2 (except ORF1ab) was used for the ELISpot assay. The samples with higher numbers of predicted TCRs for S/M/N/ORF3/ORF6 according to epitope predictions (Yh018 and Yh002) also showed relatively higher numbers of IFN-γ-producing T cells in ELISpot assays, suggesting that the results of epitope predictions should be reasonably reliable, even if individual TCR results need to be further evaluated.

The diversity of the TCR clonotypes was also estimated (Fig. 3C, D). Our results demonstrated that the diversity of T cells was generally maintained at a level similar to that in healthy controls, except for the severe Yh034 (see the section below for a further detailed analysis).

### Transcriptome profiles in the acute phase and during the clinical course in severe/critical cases

**Figure 4:**
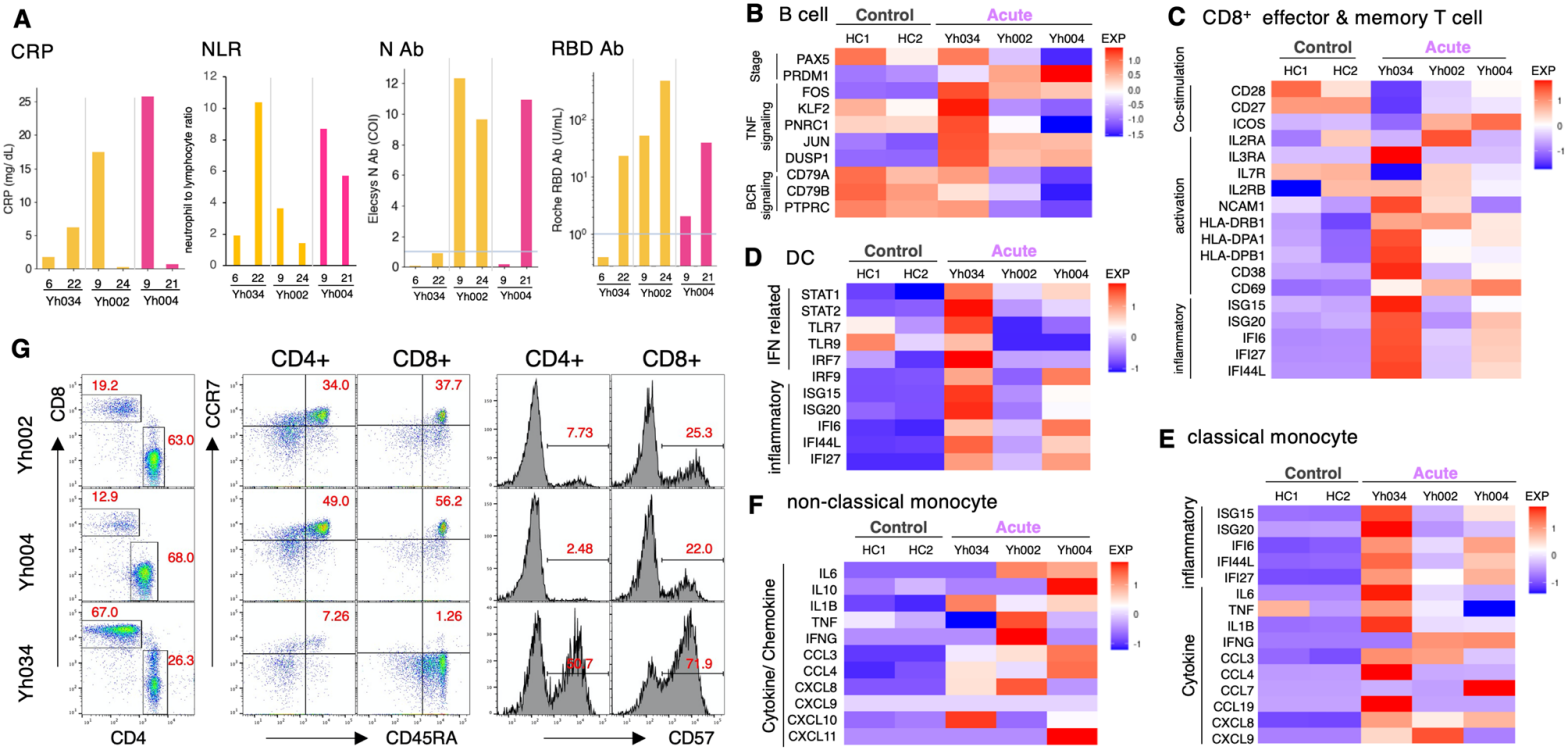
Immune profiles of severe/critical patients in acute phase.** (A)** Clinical information of Yh034, Yh002, and Yh004. CRP, NLR, and SARS-CoV-2 antibody level of the anti-RBD and anti-N regions are shown. **(B)** Expression level of development, TNF signaling, and BCR signaling activation markers in B cells. **(C)** Expression level of co-stimulation, activation, and inflammatory markers in effector and memory CD8^+^ T cells. **(D)** Expression level of IFN-γ-related and inflammatory markers in DC. **(E)** Expression level of inflammatory and cytokine-related genes in classical monocytes. **(F)** Expression level of cytokine/chemokine-related genes in non-classical monocytes. Color key for heatmap is shown in the margin.** (G)** FACS analysis.

Further detailed analyses were performed on severe/critical cases to reveal the association between the immune profile in acute phase and the clinical characteristics of COVID-19. As shown in Fig. 1B, all three patients had pneumonia and required oxygen supplementation upon hospital admission. The clinical status was severe in Yh002 and critical in Yh004 at hospital admission, and both patients were discharged 35 and 32 DPSO after viral clearance, respectively (Fig. 1B, C). In the case of Yh034, although throughout the course of infection the symptoms were not severe compared to Yh002 and Yh004, SARS-CoV-2 PCR was positive until 50 DPSO and the hospitalization was prolonged for more than 2 months (72 days) (Fig. 1C). The levels of C-reactive protein (CRP) and neutrophil-to-lymphocyte ratio (NLR), both of which are strongly associated with the severity of COVID-19^24^, were extremely high at hospital admission, the time point of single-cell analysis, but declined within a week in Yh002 and Yh004. By contrast, both increased later on 22 DPSO in Yh034 (Fig. 4A).

The cell proportion and gene expression pattern of each cell subset in the acute phase was similar in Yh002 and Yh004, while unique in Yh034. Activation- and inflammation-related genes showed higher expression in Yh034 than in Yh002/Yh004 in B cells, CD8^+^ effector and memory T cells, DC, and classical monocytes (Fig. 4B–F, Supplementary Table S3). However, the expression of IL6 and TNF, well-known markers associated with severity in COVID-19, was lower in non-classical monocytes in Yh034 than in Yh002/Yh004 (Fig. 4F), parallel to the clinical status of each patient at the time point (Fig. 1B, C). Differentially expressed genes (DEGs) analysis showed that ribosomal genes were upregulated in Yh034 (Supplementary Table S3). These data indicate that the immune status of Yh034 was hyperactivated and differed remarkably from that of other severe/critical patients.

The relative abundance of plasma cells was observed in Yh002/Yh004, but not in Yh034 (Fig. 2C), which is consistent with the gene expression pattern of B cells showing poor differentiation in Yh034 (Fig. 4B). In accordance with the B cell transcriptome profile, neither anti-RBD nor N antibodies were detected in the plasma of Yh034 at early days of infection (Fig. 4A). Though the timing of sample collection is earlier than that of Yh002/Y004, these results suggest the possibility that appropriate B cell-related immune responses were not induced in Yh034.

In the T cell subset, although the relative population of T cells was higher than that in other patients, the CD4/CD8 ratio was lower than that in other patients, and naïve CD4^+^ and CD8^+^ T cells were very rare in Yh034 (Fig. 2C). These features were confirmed by flow cytometry (Fig. 4G). The frequencies of naïve CD4^+^ and CD8^+^ T cells (CD45RA^+^CCR7^+^) were low, and most CD8^+^ T cells were terminally differentiated in Yh034 (Fig. 4G), indicating that Yh034 had much less potential to generate antigen-specific T cell responses against new pathogens. Indeed, few, if any, SARS-CoV-2-specific T-cell responses were detected in the ELISpot assay in Yh034 (Fig. 3B). Furthermore, CD57 expression, a marker of T cell senescence, was much higher in Yh034 than Yh002/Yh004 in both CD4^+^ and CD8^+^ T cells (Fig. 4G). The TCR repertoire in Yh034 also differed considerably from that in the other patients. The few expanded clonotypes occupied more than half of the T cell repertoire, and T cell diversity was extremely low compared to that in other patients (Fig. 3C, D). Yh034 was 84-years old, almost 20 years older than Yh002/Yh004 (Fig. 1C). A low CD4/CD8 ratio, decline in naïve T cells, and low TCR diversity are typical immunological features of elderly people^25^. These data suggest that the induction of both T-cell and antibody responses specific to SARS-CoV-2 are insufficient and/or delayed in Yh034 cells because of naïve T cell depletion.

Taken together, abnormal hyperactivation status, long-term inflammation, and lower SARS-CoV-2-specific immune responses may be associated with delayed viral clearance and a prolonged clinical course of COVID-19 in Yh034. These differences in immune responses may result from the individual’s background such as medical history and age.

### Longitudinal analysis in severe/critical cases

To clarify the immunological features of acute phase of COVID-19, samples from the convalescent phase of severe (Yh002) and critical (Yh004) cases were analyzed. Samples collected after the patients’ discharge were used for longitudinal analyses (Fig. 1A–C). The data obtained were processed in the same manner as for the acute phase.

**Figure 5:**
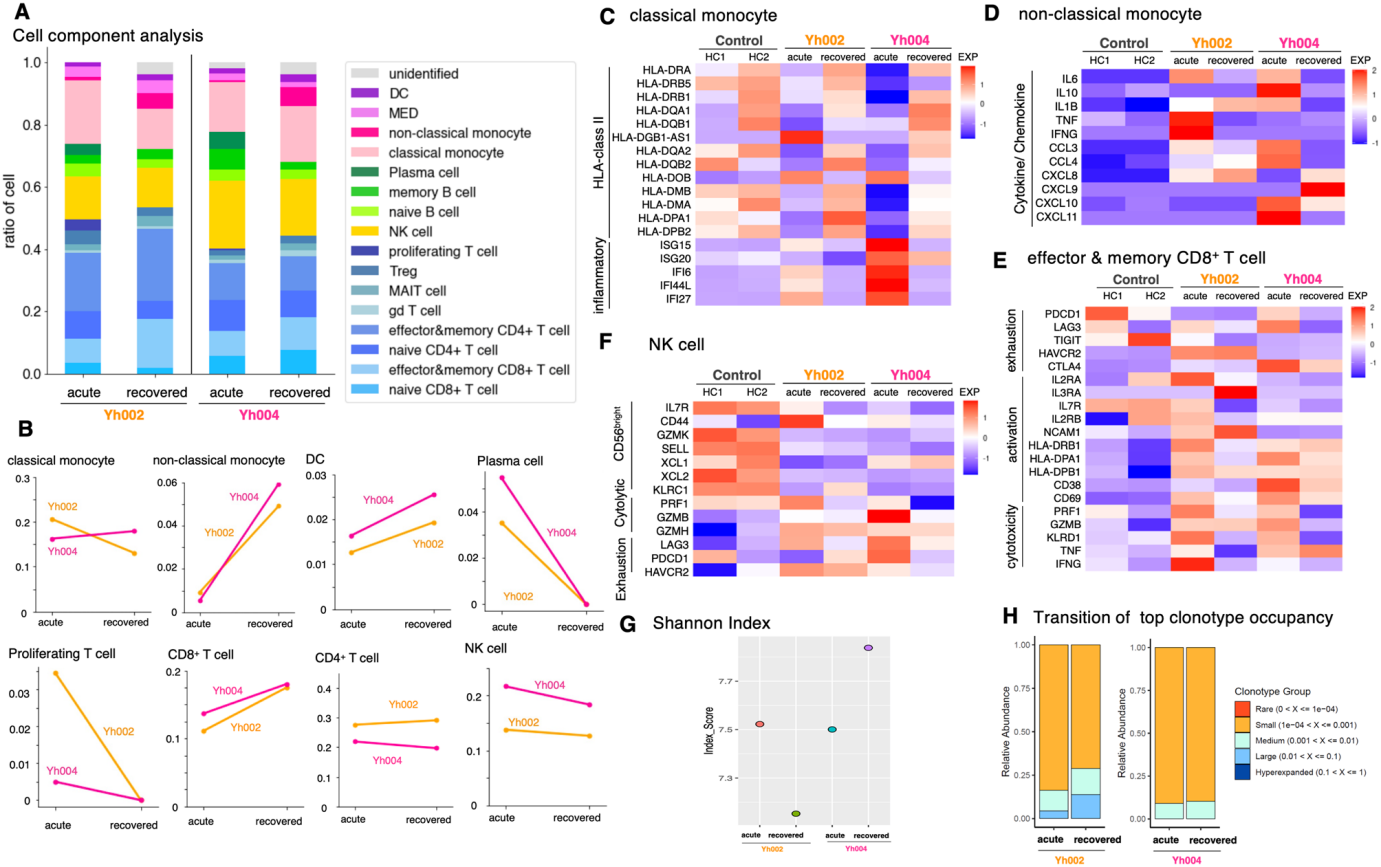
Longitudinal single-cell analysis of Yh002 (severe) and Yh004 (critical). **(A)** Cell proportion of each case. Bar plot shows cell type included in each dataset in Yh002 and Yh004. Color key is shown in the margin. **(B)** Cell proportion transition of Yh002 and Yh004 in classical monocyte, non-classical monocyte, DC, plasma cell (top, from left to right), proliferating T cell, CD8^+^ T cell, CD4^+^ T cell, and NK cell (bottom, from left to right). **(C–F)** Expression level of classical monocyte **(C),** non-classical monocyte **(D)**, effector and memory CD8^+^ T cell **(E)**, and NK cell **(F)**. **(G, H)** TCR diversity transition of Yh002 and Yh004. The *y-axis* shows the Shannon index of those patients at disease onset and after recovery from the SARS-CoV-2 infection **(G)** and clonotype group component **(H)**.

In the myeloid fraction, there was a decrease in the most abundant classical monocytes in Yh002 but not in Yh004 (Fig. 5A, B, top left panel). In both patients, inflammation-related genes were highly upregulated in the acute phase but downregulated and returned to normal levels in the convalescent phase, indicating the functional role of these innate cells in acute viral infections (Fig. 5C and Supplementary Fig. S10A). HLA-class II genes were downregulated in the acute phase, especially in the critical case Yh004, as shown in previous studies^14^, but expression levels also returned to a level similar to that of the healthy control after recovery (Fig. 5C and Supplementary Fig. S10B).

The ratio of non-classical monocytes and dendritic cells (DC) increased in the convalescent phase in both patients (Fig. 5B, top middle panel). The expression pattern of non-classical monocytes showed that cytokine/chemokine genes were upregulated during the acute infection phase in cells from both Yh002 and Yh004 (Fig. 5D and Supplementary Fig. S10C). A list of DEGs in the acute and recovered phases further demonstrated that inflammation-related genes, such as IFI27 and IFI6, were upregulated in classical and non-classical monocytes (Supplementary Table S4). These results suggest that non-classical monocytes have more specialized functions in acute antiviral responses during SARS-CoV-2 infection, as has been previously suggested in studies investigating other viral infections^26^, and may return to their original functions after the end of severe symptoms.

Plasma cells and proliferating T cells decreased in the convalescent phase (Fig. 5B, top right and bottom left). The number of these effector cells is thought to be increased by antigen-specific and/or inflammatory stimulation in the acute phase. After viral clearance and/or the reduction of the inflammatory responses, the number of these cells returns to the normal level.

The frequency of CD8^+^ T cells increased after recovery, but there was no change in CD4^+^ T cell (Fig. 5B, bottom middle panel). Gene expression of activation and cytotoxic markers of CD8^+^ effector and memory T cells was upregulated in the acute phase (Fig. 5C). The gene expression of activation markers and cytotoxic molecules (PRF1, GZMB) of T cells was upregulated in the acute phase (Fig. 5C). Regarding the CD4^+^ and CD8^+^ T cell subsets, the frequency of CD8^+^ T cells increased after recovery but that of CD4^+^ T cells did not change (Fig. 5B, bottom middle panel). Nor was there a drastic change in number of NK cells (Fig. 5B, bottom right panel). The gene expression of the activation, cytotoxic, and exhaustion markers of CD8^+^ effector and memory T cells and NK cells was upregulated in the acute phase (Fig. 5E, F). Although the expression levels in most of these genes were reduced after recovery, the expression levels of GZMB and GZMH in recovered patients were maintained at similar levels. These data suggested that it takes longer time for some genes to return to the basal expression levels after acute infection.

We also confirmed the expression pattern of NK cells, which did not show drastic changes in cell numbers (Fig. 5B, bottom right panel). Elevation of gene expression in exhaustion and cytotoxic genes was observed in the acute phase (Fig. 5F and Supplementary Fig. S10D). Analysis of DEGs in NK cells and CD8^+^ effector and memory T cells shows an upregulation of genes related to cytotoxicity and cytokine release, such as KLRF1, KLRD1, and PRF1 (Supplementary Table S4).

Longitudinal changes in the TCR repertoire were also investigated. TCR diversity, as calculated by the Shannon index, decreased in Yh002 but increased in Yh004 (Fig. 5G). The populations of expanded TCR groups showed consistent and significant increases in Yh002 (see the “medium” and “large” clonotype groups; Fig. 5H).

In summary, these data suggest that most immune cell subsets are activated in response to the acute phase of SARS-CoV-2 infection, and the activation status generally returns to normal levels a month after symptom onset, although it may take more time for some specific genes to return to normal.

## Discussion

In this study, we conducted PBMC profiling of COVID-19 patients to determine their overall cellular composition, gene expression, and TCR repertoire. Overall, the observed molecular features were consistent with those described in recent studies worldwide. However, we also identified several substantial latent diversities between individuals, which may be associated with different clinical outcomes. Further in-depth understanding of the distinct immune landscapes of different individuals, which would have been shaped by their previous medical histories or exposure to certain environments, can help medical professionals design effective personalized therapeutic strategies for COVID-19 patients.

Analysis of COVID-19 patients, including longitudinally collected samples, revealed that the immune response to SARS-CoV-2 differs depending not only on severity but also on individual backgrounds. Our dataset provides a fundamental resource for optimizing the clinical treatment of COVID-19 patients.

## Methods

### Blood collection from patients

Six patients and two healthy controls were included in the study. First, blood samples from the patients were collected on the day of or one day after hospitalization, before treatment. Overall clinical symptoms of the patients were; mild/moderate (Yh015, Yh018 and Yh017) and severe/critical (Yh034, Yh002 and Yh004). The disease severity was categorized using chest computed tomography, X-ray, and clinical care (Fig. 1A–C). “Mild” disease was defined as asymptomatic, or symptomatic without pneumonia. “Moderate” and “severe” disease were defined by the presence of pneumonia without or with the need for supplemental oxygen, respectively. Critical disease was defined as the need for ICU admission and/or mechanical ventilation.

### 10 × Genomics Chromium scRNA-seq and scVDJ-seq of T cell

To acquire the scRNA-seq (single-cell RNA sequencing) and scVDJ (single-cell T cell receptor sequencing) library, we followed the User Guide’s instructions (10 × Genomics, USER Guide Chromium Next GEM Single Cell V(D)J, Reagent Kit v1.1, CG000207 Rev E). The target recovery number was 8000 cells. After library construction, GEX libraries were sequenced using NovaSeq (statistics for NGS are summarized in Table S1).

### Data analysis of scRNA-seq using cell ranger and R package Seurat

Output files were produced using the 10 × Genomics Cell Ranger pipeline. The following reference and parameters were used: hg38, –expected cell count, 8000. The output files were then processed by the R package Seurat^27^ (version 3.2) as a vignette. To remove low-quality datasets, we utilized the threshold nFeature_RNA > 1000, nFeature < 5000 and percent.mt < 25. After filtering, the cells were clustered and visualized using Uniform Manifold Approximation and Projection, UMAP. Each cluster was first roughly annotated as myeloid (CD14 and FCGR3A), B cells (CD19 and MS4A1), NK cells (NCAM1 and NKG7), T cells (CD3D, CD8A, and CD4), and “unidentified” based on previously reported representative markers (Figs. S1, S2 and S6). For detailed cell-type annotation, the cells were clustered and annotated again. Myeloid were divided into classic monocytes (CD14), non-classic monocytes (FCGR3A aka CD16), MED (CD14 and FCGR3A), and DC (CD1C, CLEC4C, and CLEC9A). B cells were divided into naïve B cells (IL4R and TCL1A), memory B cells (CD19, MS4A1, CD79A, and CD79B), and plasma cells (SDC1, XBP1, and MZB1). T cells were divided into naïve CD8^+^ T cells (CD8A, CCR7), memory and effector CD8^+^ T cells (CD8A), naïve CD4^+^ T cells (CD4, CCR7), memory and effector CD8^+^ T cells (CD8A), Tregs (FOXP3), gd T cells (TRGV9), MAIT (TRAV1-2), and proliferating T cells (CD3D, MKI67) (Figs. S3 and S5).^28^. The Seurat command “Dotplot” was utilized to generate the dotplots shown in Figs. S3 and S5. The Seurat “Findmarker” and “Findallmarkers” functions with their default settings were used for the statistical tests. P-values were calculated using the Wilcoxon Rank Sum test.

#### Heatmap for scRNA-seq datasets

To plot the heatmap, Seurat “AverageExpression” and “DoHeatmap” commands were employed following Seurat’s vignette. The gene list used in the heatmaps is as follows. For inflammatory: ISG15, ISG20, IFI6, IFI44L, IFI27; for HLA-class II: HLA-DRA, HLA-DRB5, HLA-DRB1, HLA-DQA1, HLA-DQB1, HLA-DQB1-AS1, HLA-DQA2, HLA-DQB2, HLA-DOB, HLA-DMB, HLA-DMA, HLA-DOA, HLA-DPA1, HLA-DPB2; for cytokines/chemokines: IL6, IL10, IL1B, IL1B, TNF, IFNG, CCL3, CCL4, CXCL8, CXCL9, CXCL10, and CXCL11; for NK cells: CD56 ^bright^, IL7R, CD44, GZMK, SELL, XCL1, XCL2 and KLRC^29^; for cytolytic molecules found in NK cells: PRF1, GZMB, and GZMH^29^; for exhaustion of NK cells: LAG3, PDCD1, and HAVCR2; for exhaustion of T cells: PDCD1, LAG3, TIGIT, HAVCR2, and CTLA4^30^; for activation of T cells: IL2RA, IL3RA, IL7R, IL2RB, NCAM1, HLA-DRB1, HLA-DPA1, HLA-DPB1, CD38, and CD69^30^; for cytotoxicity in T cells: PRF1, GZMB, KLRD1, TNF, and IFNG; for the B-cell differentiation stage: PAX5 and PRDM1; for T-cell co-stimulation: CD28, CD27, and ICOS^30^; for B cell TNF signaling: FOS, PNRC1, JUN, and DUSP1^15^; for B cell BCR signaling activation: CD79A, CD79B and PTPRC^15^; and for the IFN-related genes in DC: STAT1, STAT2, TLR7, TLR9, IRF7, and IRF9^31^.

#### Data analysis of single-cell TCR

Output files were produced using 10 × Genomics Cell Ranger pipeline and then processed by the R package scRepertoire^32^ (version 1.0.0) as a vignette. Using the datasets acquired by scRepartoire, the CDR3_aa1, CDR3_aa2, V, and J gene datasets were analyzed using TCRex^23^ (version 2020–10-28).

#### PBMC dataset from age-controlled healthy individual

For the age-controlled analysis shown in Supplementary Fig. S8, we used a dataset from a previously published study^15^. The dataset is derived from Human Cell Atlas (https://data.humancellatlas.org/). Among healthy donors, BCGV12, 70 y.o., sex unknown, disease status: normal, was selected as the control for our current dataset.

#### ELISA

Antibody titers for RBD and N were measured by cobas e411 (roche) according to the manufacturer’s instructions. Elecsys Anti-SARS-CoV-2 S RUO kit (roche) was used for the RBD antibody, and Elecsys Anti-SARS-CoV-2 RUO kit (roche) was used for the N antibody.

#### Interferon gamma (IFN-γ) ELISpot assay

The IFN-γ ELISpot assay was performed as previously described^33^. Overlapping peptide (OLP) pools spanning SARS-CoV-2 S, N, M, E, ORF3a/6/7a/7b/8/9b/10 proteins were used for antigen stimulation at 100 ng/mL of each OLP (JPT). In addition, cells were incubated in a medium without peptides as a negative control (Supplemental Figure S11).

#### Flow cytometry

The following antibodies were used for the flow cytometric analysis: anti-CD57-FITC, anti-CD28-PE, anti-CD4-PerCP, anti-CD8α-PerCP, anti-CCR7-PE-Cy7, anti-CD27-APC, anti-CD3-APC-Cy7, anti-CD45RA-APC-Cy7, anti-CD3-Pacific Blue, and anti-CD3-Pacific Blue (BioLegend, U.S.). A LIVEDEAD™ Fixable Aqua Dead Cell Stain kit (Thermo Fisher Scientific, U.S.) was used to monitor the cell viability. All flow data were acquired using FACS Canto II (Becton Dickinson), and the analysis was performed using FlowJo software ver.10.8.0 (Tree Star, U.S.).

### Approval for human experiments

The patients and healthy donor PBMC used in the current study were approved by the NIID ethics committee (examination number: 1197) and the University of Tokyo ethics committee (examination number: 20-351). COVID-19 patients were enrolled at Yokohama Municipal Citizen’s Hospital between March 2020 and May 2020. All methods were carried out in accordance with relevant guidelines and regulations. Informed consent was obtained from all participants enrolled in the current study.

## Supplementary Information

Supplementary Information 1.

Supplementary Information 2.

## Data Availability

For control samples HC1 and HC2, we utilized our previous study dataset, the raw datasets of which (count matrix) were deposited in the National Bioscience Database Center (study number: JGAS000321). For other scRNA-seq and scVDJ-seq datasets, we deposited the raw datasets in the National Bioscience Database Center (study number: JGAS000546). The present study did not develop any new software.
