# Supplementary material for: Multimodal single-cell analyses of peripheral blood mononuclear cells of COVID-19 patients in Japan
Source: Sci Rep. 2023 Feb 2;13:1935. doi: 10.1038/s41598-023-28696-9 (PMC9893982; doi:10.1038/s41598-023-28696-9)
Supplement: Supplementary file 1 — Supplementary Information 1. [file 41598_2023_28696_MOESM1_ESM.pdf]

Supplementary Figures

Multimodal single-cell analyses of peripheral blood mononuclear cells of COVID-19 patients in Japan

Yukie Kashima[1], Taketoshi Mizutani[1], Kaori Nakayama-Hosoya[2], Saya Moriyama[3], Takayuki Matsumura[3], Yoshihiro Yoshimura[4], Hiroaki Sasaki[4], Hiroshi Horiuchi[4], Nobuyuki Miyata[4], Kazuhito Miyazaki[4], Natsuo Tachikawa[4], Yoshimasa Takahashi[3], Tadaki Suzuki[5], Sumio Sugano[6], Tetsuro Matano[2,7,8], Ai Kawana-Tachikawa[2,7,8], Yutaka Suzuki[1,*]

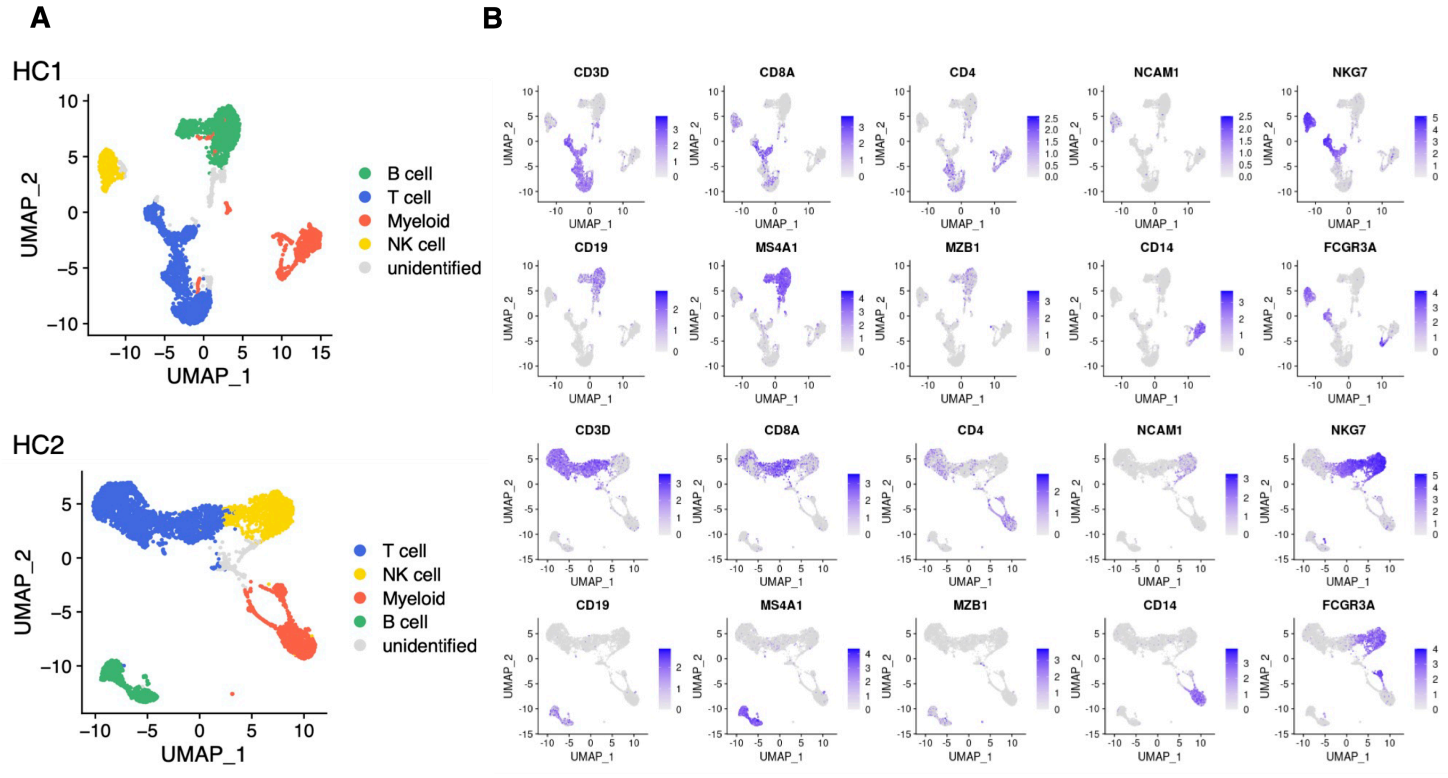

Supplementary Fig S1. Cell annotation of scRNA-seq datasets of healthy controls
(A) Annotation of scRNA-seq datasets of HC1 and HC2. UMAP colored by sample origin. Color key is shown in the margin. **(B)** Feature plots of representative markers: CD3, CD8A, and CD4 (T cell), NCAM1 and NKG7 (NK cell), CD19, MS4A1, and XBP1 (B cell and Plasma cell), CD14 and FCGR3A (myeloid).

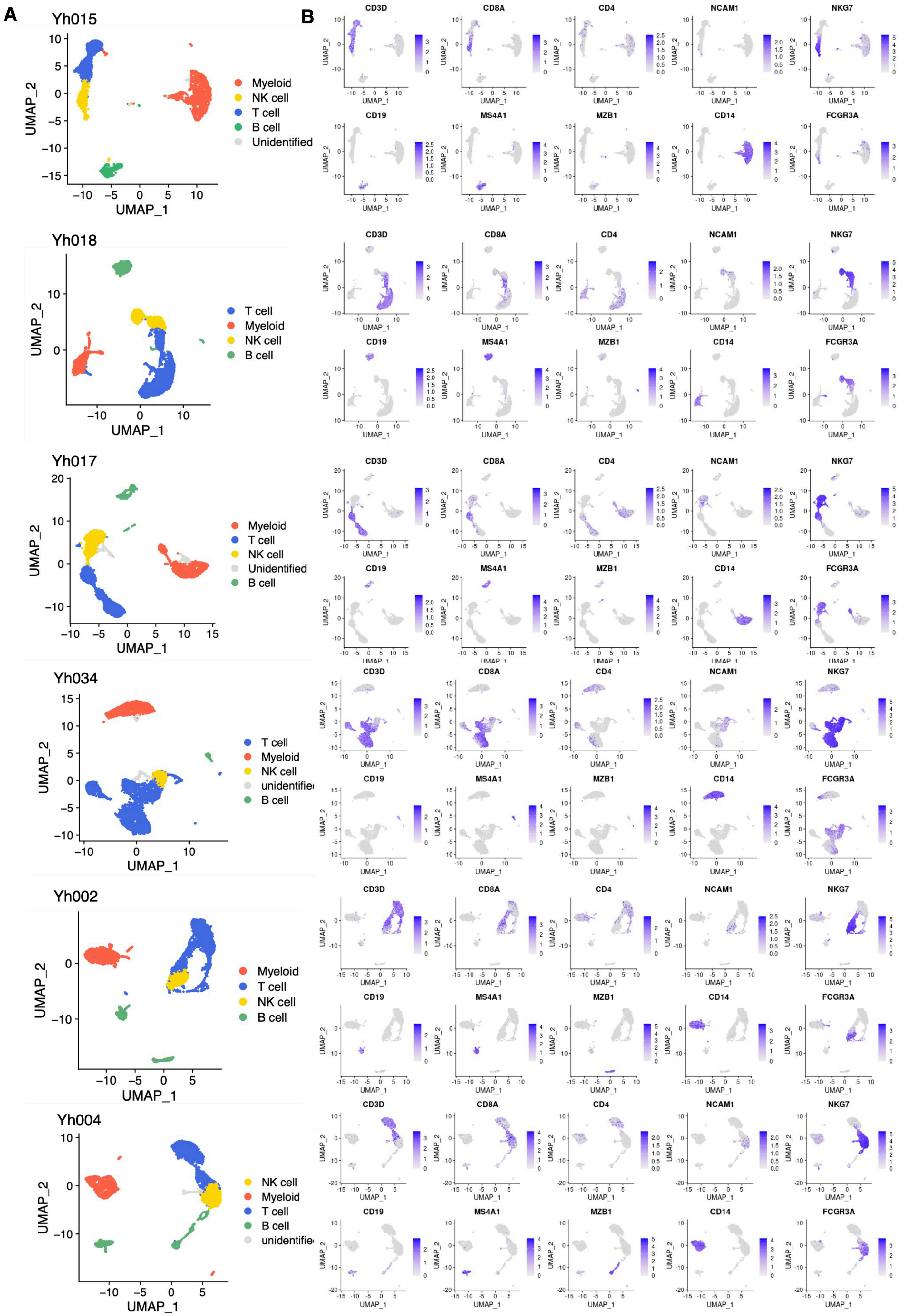

Supplementary Figure S2

Supplementary Fig S2. Cell annotation of scRNA-seq datasets of COVID-19 patients

(A) Annotation of scRNA-seq datasets of HC1 and HC2. UMAP colored by sample origin. Color key is shown in the margin. **(B)** Feature plots of representative markers: CD3, CD8A, and CD4 (T cell), NCAM1 and NKG7 (NK cell), CD19, MS4A1, and XBP1 (B cell and Plasma cell), CD14 and FCGR3A (myeloid).

Annotation of Yh002 post and Yh004 post

A **B**

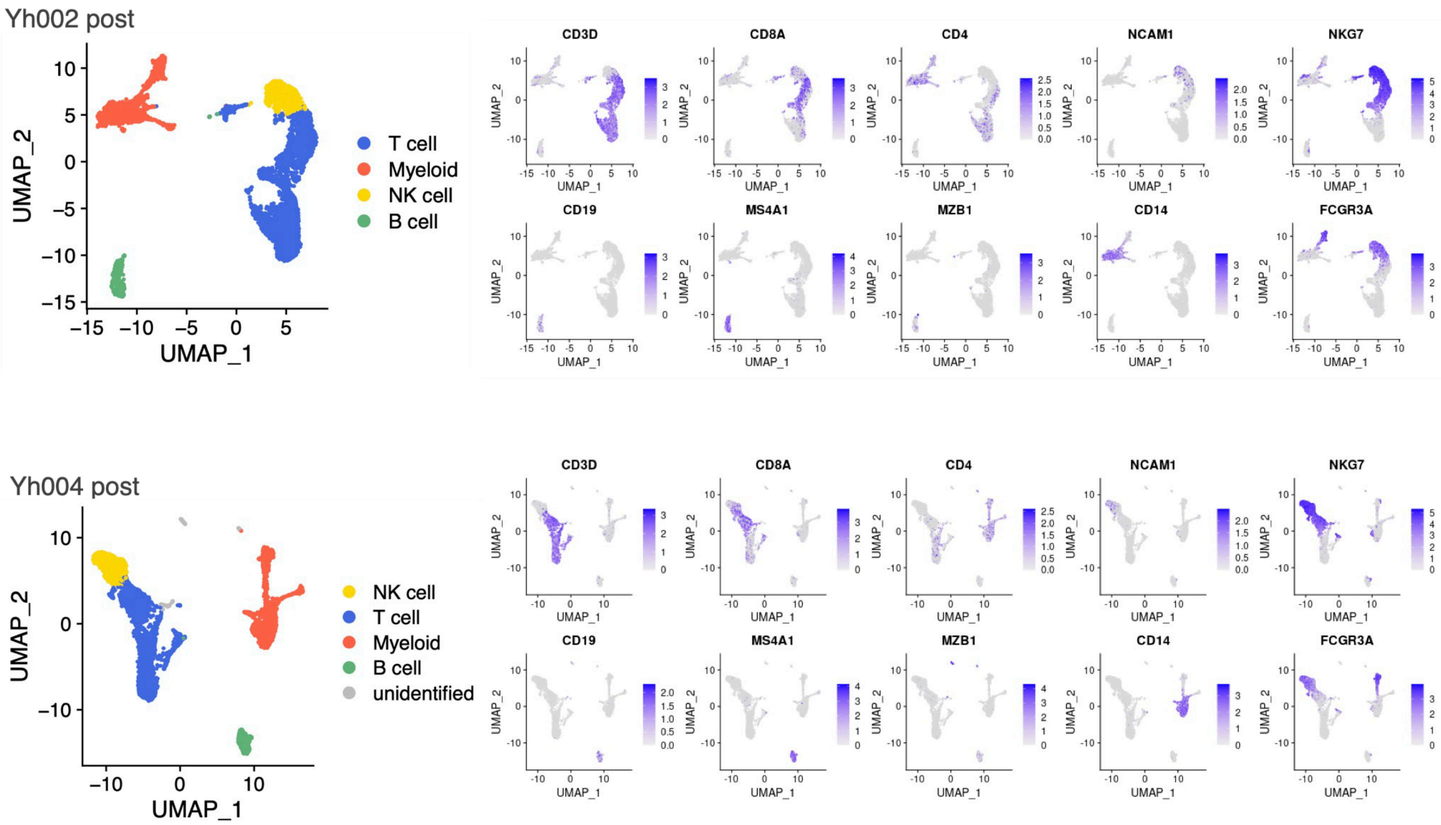

Supplementary Fig S3. Cell annotation of scRNA-seq datasets of Yh002 and Yh004 in the convalescent phase

(A) Annotation of scRNA-seq datasets of HC1 and HC2. UMAP colored according to sample origin. Color key is shown in the margin. **(B)** Feature plots of representative markers: CD3, CD8A and CD4 (T cell), NCAM1 and NKG7 (NK cell), CD19, MS4A1 and XBP1 (B cell and Plasma cell), CD14 and FCGR3A (myeloid).

Annotation of severity study samples

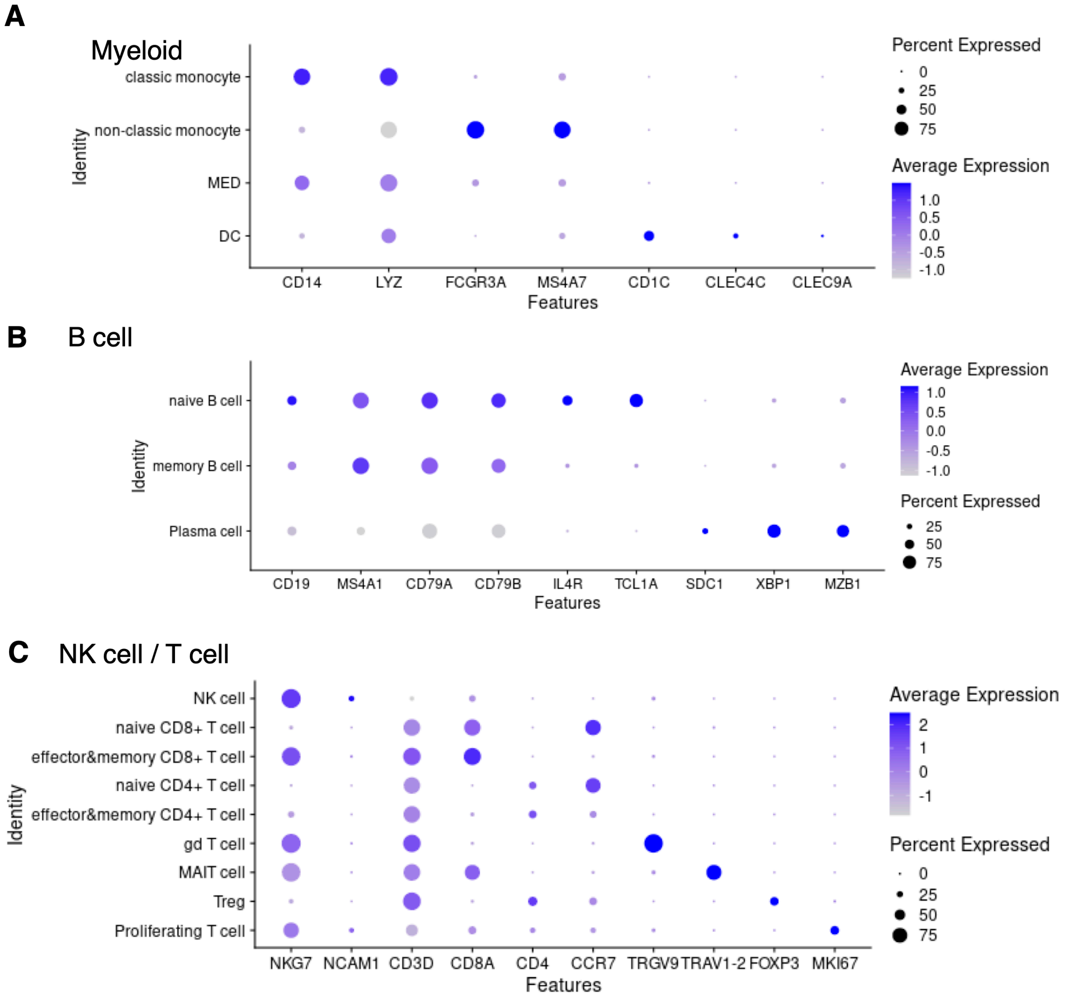

Supplementary Fig S4. Expression of representative markers of severity study samples
(A- C) Expression level of representative markers for myeloid **(A)**, B cell **(B)**, NK cell, and T cell **(C)** are shown as dotplot. Samples used in the severity study, HC1, HC2, Yh015, Yh018, Yh017, Yh034, Yh002, and Yh004, are included. Legend is shown in the margin. Detailed information of used markers is shown in the material and method section.

Annotation of longitudinal study samples

A Myeloid

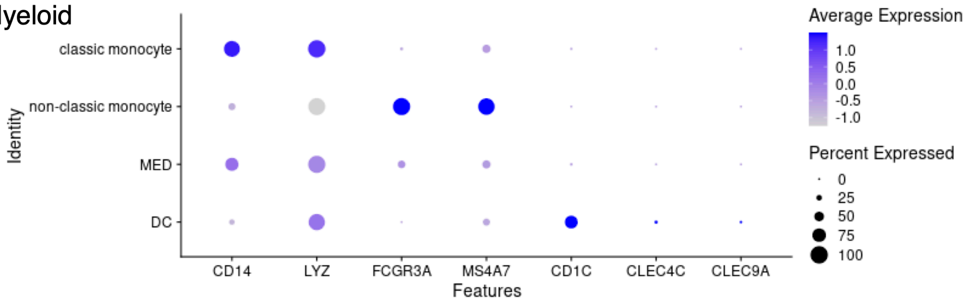

B B cell

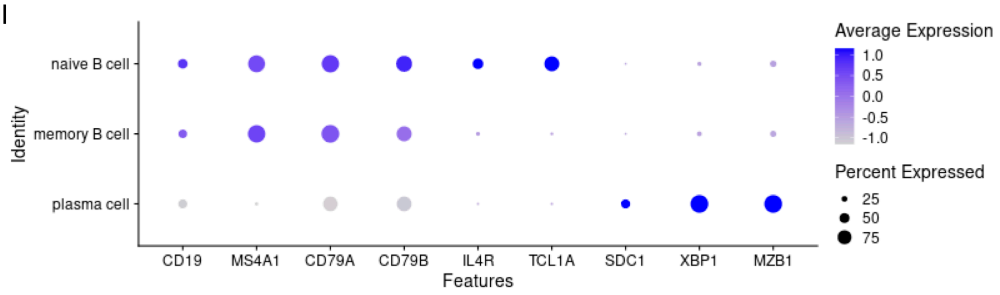

C NK cell / T cell

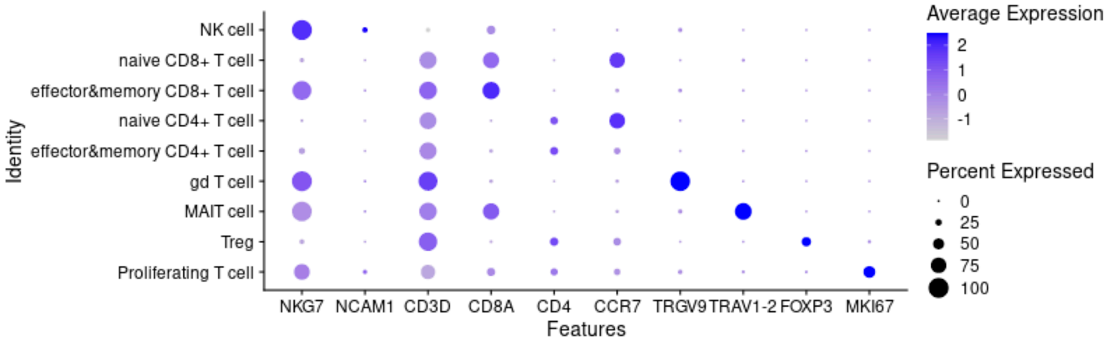

Supplementary Fig S5. Expression of representative markers of longitudinal study samples
(A- C) Expression level of representative markers for myeloid **(A)**, B cell **(B)**, NK cell, and T cell **(C)**. Samples used in the longitudinal study, HC1, HC2, Yh002, Yh002post, Yh004, and Yh004post, are included. Legend is shown in the margin. Detailed information of used markers is shown in the material and method section.

UAMP including all samples used in the current study

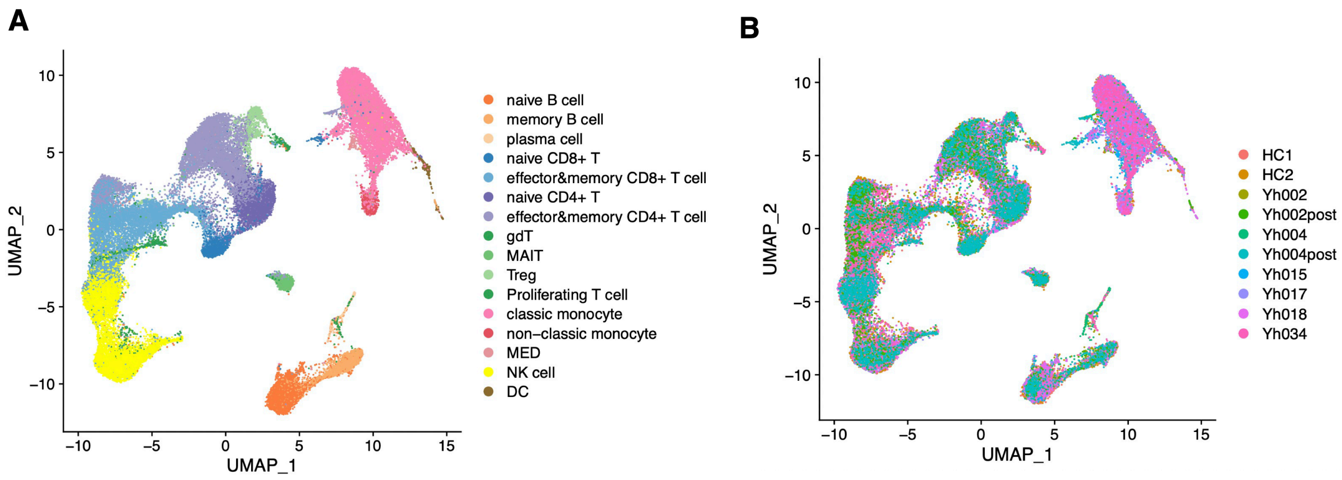

Supplementary Fig S6. UAMP including all samples used in the current study
(A- B) UMAP of all 10 samples (HC1, HC2, Yh002, Yh004, Yh015, Yh017, Yh018, Yh034, Yh002post, and Yh004post) included in the current study. UMAP is colored by cell type **(A)** and cell origin **(B)**. Color key is shown in the margin.

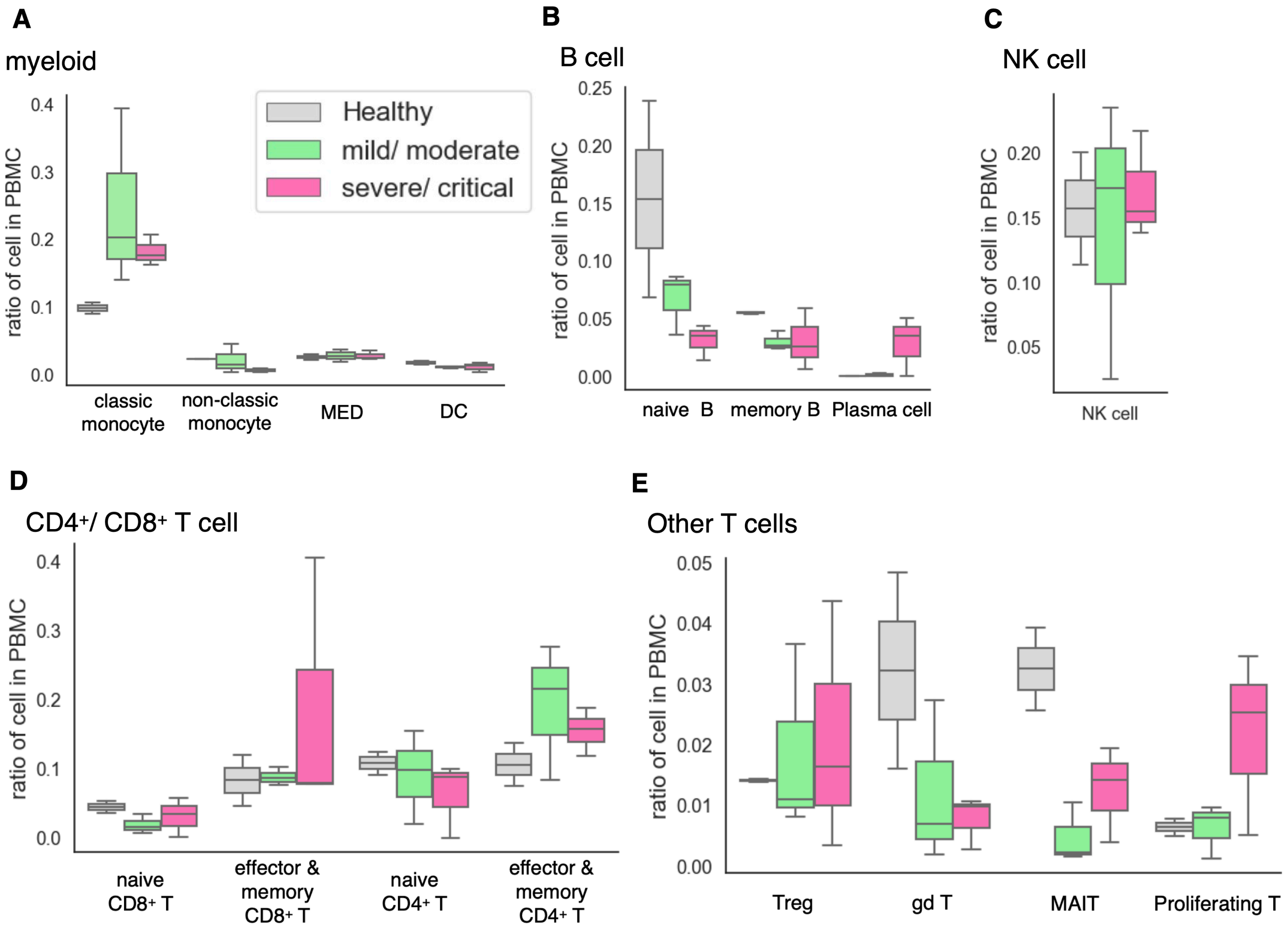

Supplementary Fig S7. Comparison of cell proportion in healthy controls, mild/moderate, and severe/critical patients

(A- E) Box plot showing each cell type ratio. Myeloid fraction; classical monocyte, non-classical monocyte, MED, and DC **(A)**, B cell fraction; naïve B cell, memory B cell, and Plasma cell **(B)**, NK cell **(C)**, naïve CD8+ T cell, effector and memory CD8+ T cell, naïve CD4+ T cell, and effector and memory CD4+ T cell **(D)**, Treg, gd T cell, MAIT cell, and proliferating T cell **(E)**.

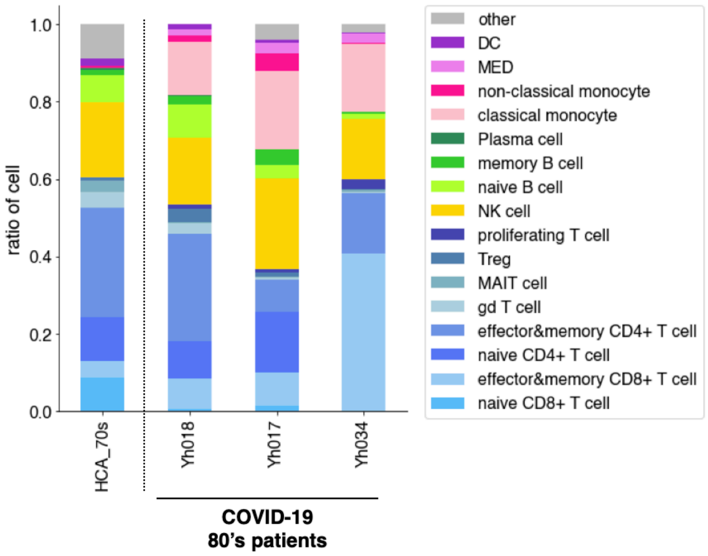

Supplementary Fig S8. Comparison of cell proportions in older healthy controls and COVID-19 patients

Bar plot shows cell proportions of each donor. One healthy age-controlled donor (70 y.o. disease status: normal) and three COVID-19 patients (Yh018: 86 y.o., Yh017: 85 y.o., and Yh034: 84 y.o.) are included. Cells were grouped from the datasets into naïve CD8+ T cell, effector and memory CD8+ T cell, naïve CD4+ T cell, effector and memory CD4+ T cell, gd T cell, MAIT cell, Treg cell, proliferating T cell, NK cell, naïve B cell, memory B cell, plasma cell, classical monocyte, non-classical monocyte, MED, DC, and other. Color key is shown in the margin.

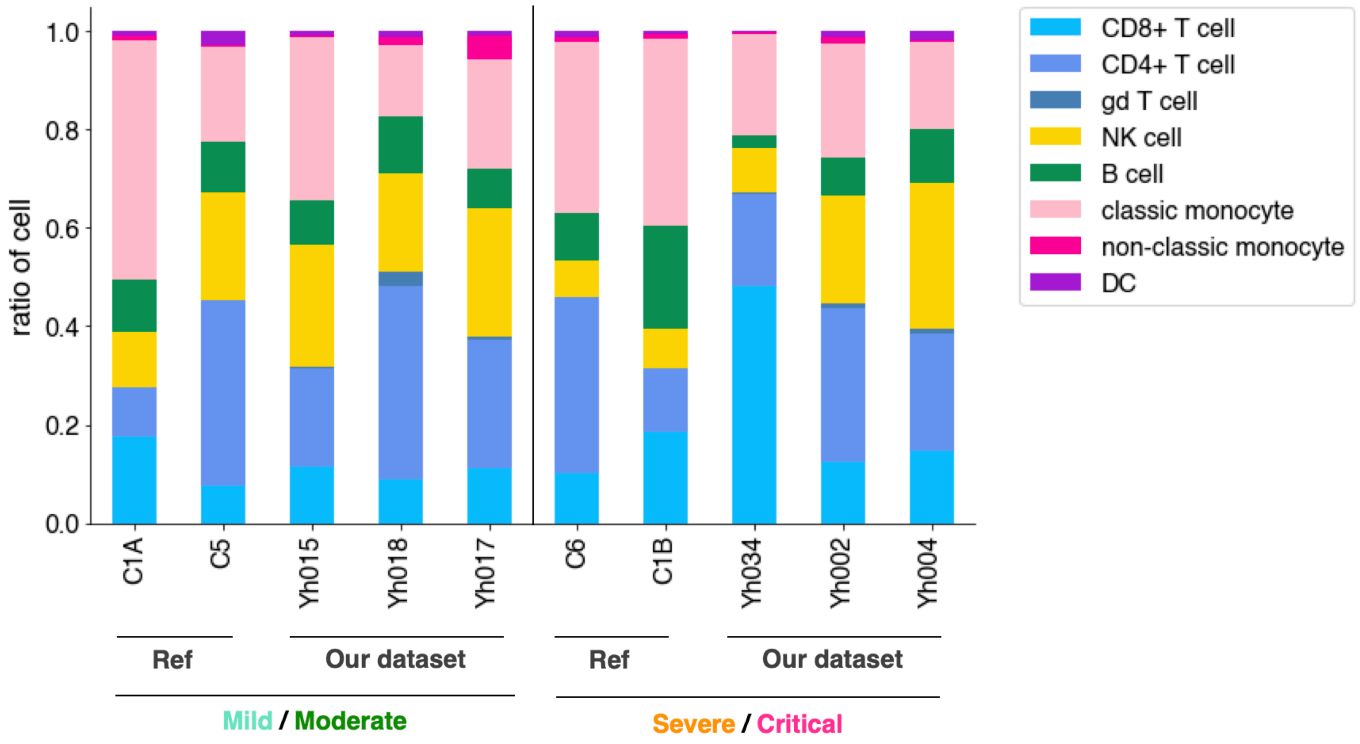

Supplementary Fig S9. Comparison of our current datasets and previously reported COVID-19 patients

Bar plot shows cell proportions of each donor. Ref shows previously reported dataset. We compared datasets by forming the following groups: CD8+ T cell, CD4+ T cell, gd T cell, NK cell, B cell, classical monocyte, non-classical monocyte, and DC. Color key is shown in the margin.

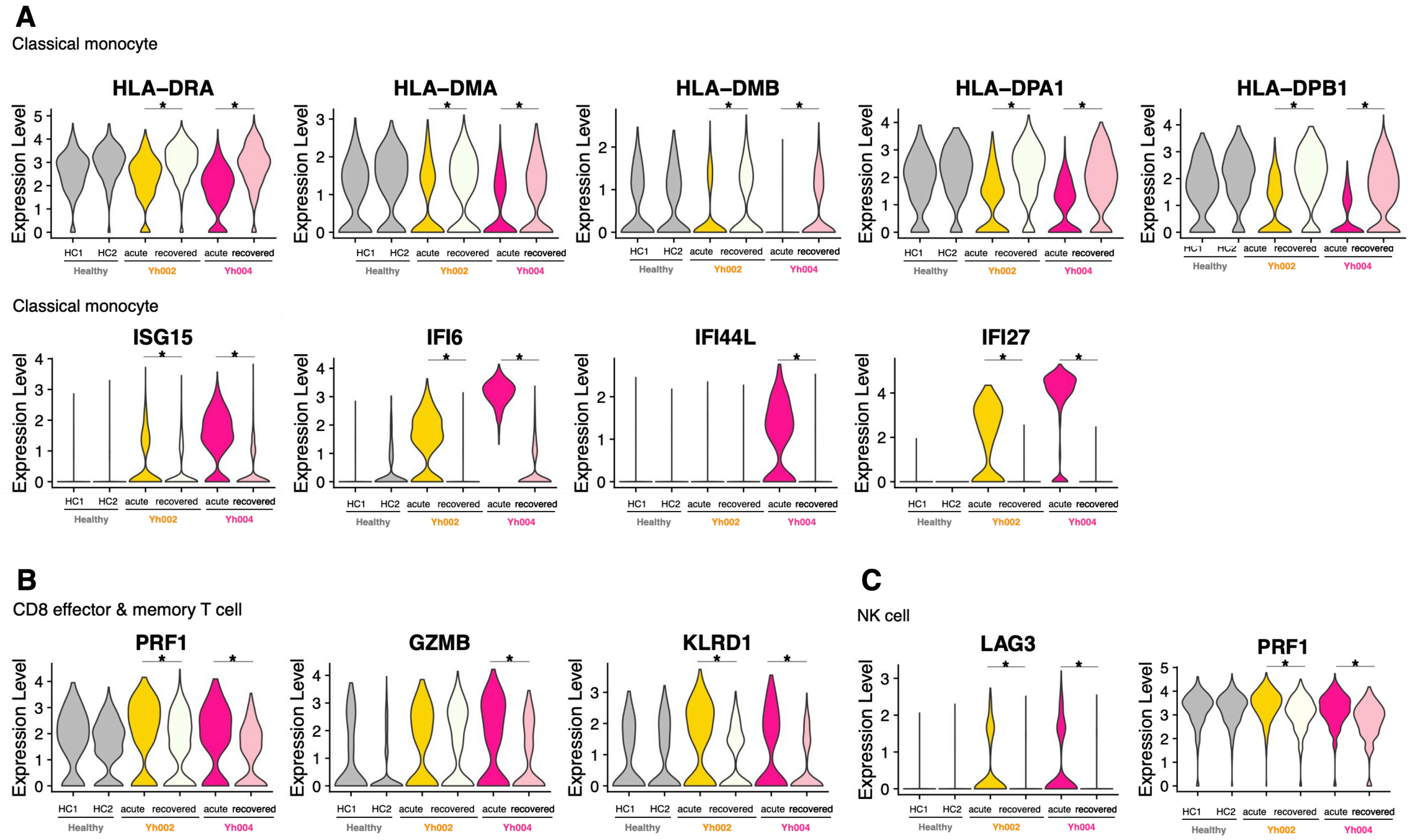

Supplementary Fig S10. Comparison of our current datasets and previously reported COVID-19 patients

(A- C) Expression level of representative marker genes in classical monocytes **(A)** CD8+ effector and memory T cell **(B)**, and NK cell **(C)**. Statistical differences were tested for in the acute infection phase and after recovery in Yh002 and Yh002 respectively and significant differences are marked with * ($p < 0.05$, Wilcoxon Rank Sum test).

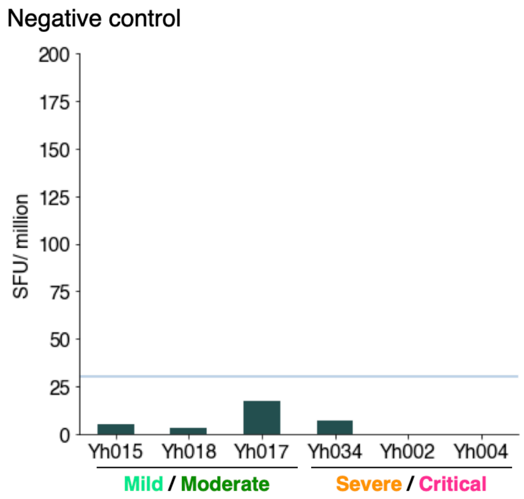

Supplementary Fig S11. ELISpot Assay control
Bar plot showing the result of ELISpot assay of the negative control. (Results for target peptides are shown in Figure 3). Gray line shows the threshold (30 SFC/ million PBMCs).
