# Supplementary material for: Multimodal single-cell analyses of peripheral blood mononuclear cells of COVID-19 patients in Japan
Source: Sci Rep. 2023 Feb 2;13:1935. doi: 10.1038/s41598-023-28696-9 (PMC9893982; doi:10.1038/s41598-023-28696-9)
Supplement: Supplementary file 2 — Supplementary Information 2. [file 41598_2023_28696_MOESM2_ESM.pdf]

Supplementary Tables

Multimodal single-cell analyses of peripheral blood mononuclear cells of COVID-19 patients in Japan

Yukie Kashima[1], Taketoshi Mizutani[1], Kaori Nakayama-Hosoya[2], Saya Moriyama[3], Takayuki Matsumura[3], Yoshihiro Yoshimura[4], Hiroaki Sasaki[4], Hiroshi Horiuchi[4], Nobuyuki Miyata[4], Kazuhito Miyazaki[4], Natsuo Tachikawa[4], Yoshimasa Takahashi[3], Tadaki Suzuki[5], Sumio Sugano[6], Tetsuro Matano[2,7,8], Ai Kawana-Tachikawa[2,7,8], Yutaka Suzuki[1, *]

Supplementary Tables

Supplementary Table S1 Detailed sequence statics in the current study
Metadata for the scRNA-seq datasets used in this study.

Supplementary Table S2 SARS-CoV-2 specific T cell in early onset patients
T cells predicted to be SARS-CoV-2, specified by TCRex.

Supplementary Table S3 DEGs between Yh002/Yh004 and Yh034
DEGs compared between Yh034 and Yh002/Yh004. Top 20 DEGs of B cell, DC, classical monocyte, non-classical monocyte, and CD8+ memory and effector T cell upregulated in Yh034 are shown.

Supplementary Table S4 DEGs between COVID-19 acute and recovered phase samples
A comparison of DEGs in the acute phase (Yh002, Yh004) and recovered phase (Yh002post, Yh004post). Top 20 DEGs of NK cell, classical monocyte, non-classical monocyte, and CD8+ memory and effector T cell are shown.

Table S1 Sequence Statistics of the current study

case ID	severity	scGEX			scVDJ		
		total reads	% >= Q30 bases	cell number	total reads	% >= Q30 bases	cell number
**HC_1**	**-**	531,399,627	88.60	4,591	103,472,914	93.40	2,092
**HC_2**	**-**	462,214,322	88.30	5,191	118,607,055	94.30	2,924
**Yh015**	**Mild**	352,287,834	90.60	3,305	125,905,586	92.59	1,546
**Yh018**	**Mild**	465,335,858	90.47	5,960	124,245,056	93.61	3,380
**Yh017**	**Moderate**	507,000,922	91.28	4,731	138,209,961	94.29	2,336
**Yh034**	**Severe**	463,301,946	90.91	8,396	115,217,608	93.05	4,343
**Yh002**	**Severe**	433,665,581	88.64	4,083	101,867,007	91.93	2,988
**Yh002post**	**convalescent**	517,457,803	89.88	5,285	112,578,673	91.87	3,599
**Yh004**	**Critical**	440,967,013	89.57	3,603	98,569,184	91.58	2,202
**Yh004post**	**convalescent**	507,410,804	88.55	6,272	131,426,415	92.17	3,487

Table S2 List of T cells predicted to SARS-CoV-2 in donors

CellBarcode	TRBV	TRBJ	CDR3	gene
HC1_AACTTTCGTGTTGAGG-1	TRBV29-1	TRBJ2-7	CSVGDGSYEQYF	ORF1ab
HC1_AATCGGTTCAGCTGGC-1	TRBV2	TRBJ2-7	CASSDRGYEQYF	ORF1ab
HC1_ACCGTAAAGGGCATGT-1	TRBV7-9	TRBJ2-3	CASSRDISTDTQYF	S(ORF2)
HC1_ACGCCAGAGGTGGGTT-1	TRBV28	TRBJ2-7	CASSLGSSYEQYF	ORF1ab
HC1_AGAGCTTTCGTTGCCT-1	TRBV9	TRBJ2-2	CASSIGGGELFF	ORF1ab
HC1_AGATTGCTCCAAGTAC-1	TRBV5-1	TRBJ2-7	CASSFGGSYEQYF	ORF1ab
HC1_AGCGGTCCAGAGCCAA-1	TRBV5-4	TRBJ2-2	CASSVGQNTGELFF	ORF1ab
HC1_AGGGTGACAGACAAAT-1	TRBV12-3	TRBJ2-7	CASSLGQGYEQYF	ORF1ab
HC1_AGGGTGATCCTTGACC-1	TRBV29-1	TRBJ2-7	CSVGDGSYEQYF	ORF1ab
HC1_AGTAGTCTCCAAACTG-1	TRBV7-3	TRBJ2-1	CASSLLAGGYNEQFF	ORF1ab
HC1_ATTATCCAGTGTTAGA-1	TRBV13	TRBJ2-3	CASSLGGDTQYF	N(ORF9)
HC1_CACAAACTCGCTGATA-1	TRBV7-9	TRBJ2-5	CASSLAGEETQYF	S(ORF2)
HC1_CAGTCCTGTGTGTGCC-1	TRBV5-1	TRBJ2-7	CASSPDRGLSYEQYF	ORF1ab
HC1_CATATGGTCGCACTCT-1	TRBV18	TRBJ2-7	CASSPGYEQYF	ORF1ab
HC1_CCGTTCACATGATCCA-1	TRBV7-2	TRBJ2-1	CASSLVVAGGQNEQFF	ORF1ab
HC1_CCTACCAAGGAGCGTT-1	TRBV4-1	TRBJ2-3	CASSQDDRATDTQYF	ORF1ab
HC1_CGGACGTCACCTCGGA-1	TRBV3-1	TRBJ2-5	CASSQEGADQETQYF	ORF1ab
HC1_CGTGTAAAGTCGCCGT-1	TRBV5-5	TRBJ2-7	CASSSGTGDYEQYF	S(ORF2)
HC1_GATCGCGTCTTTACAC-1	TRBV7-2	TRBJ2-7	CASSLGTGGSYEQYF	ORF1ab
HC1_GCATGATAGTGACTCT-1	TRBV28	TRBJ2-7	CASSYGYEQYF	N
HC1_GCGAGAAGTTAAGACA-1	TRBV14	TRBJ2-7	CASSSTGYEQYF	ORF1ab
HC1_GGACAAGGTTCGGGCT-1	TRBV7-7	TRBJ2-7	CASSLDSSYEQYF	ORF1ab
HC1_GGAGCAAAGTGGAGTC-1	TRBV7-9	TRBJ2-7	CASSFSYEQYF	ORF1ab
HC1_GTATTCTAGTGGTCCC-1	TRBV4-1	TRBJ2-6	CASSQDQSGANVLTF	ORF1ab
HC1_GTCACGGGTCACCTAA-1	TRBV7-6	TRBJ1-1	CASSLGGSTEAFF	N
HC1_GTTAAGCGTGCGCTTG-1	TRBV5-1	TRBJ2-7	CASSVGQGSYEQYF	S(ORF2)
HC1_GTTTCTACATGTCTCC-1	TRBV6-4	TRBJ2-1	CASSELAGGLNGEQFF	ORF3a
HC1_TACTCGCAGGCATTGG-1	TRBV12-3	TRBJ2-3	CASSFGTDTQYF	ORF1ab
HC1_TTCTCAAGTCGATTGT-1	TRBV12-3	TRBJ2-2	CASSLGTGELFF	ORF1ab
HC2_AAGGTTCAGCTTCGCG-1	TRBV9	TRBJ2-1	CASSGDGYNEQFF	ORF1ab
HC2_ACACCAACACCACCAG-1	TRBV6-1	TRBJ2-1	CASSEAGLANEQFF	ORF1ab
HC2_ACGGAGAAGTTTAGGA-1	TRBV12-3	TRBJ1-1	CASSWGGNTEAFF	ORF1ab
HC2_ACGTCAAAGCTAGGCA-1	TRBV13	TRBJ2-2	CASSLVSGELFF	ORF1ab
HC2_AGTCTTTTCATTGCGA-1	TRBV4-1	TRBJ2-1	CASSQDLALSSYNEQFF	ORF1ab
HC2_ATGAGGGTCCGCTGTT-1	TRBV9	TRBJ2-7	CASSSSGYEQYF	ORF1ab
HC2_CAAGATCCATTACCTT-1	TRBV7-9	TRBJ1-1	CASSLGLNTEAFF	ORF6
HC2_CAGCCGAAGCCTATGT-1	TRBV7-9	TRBJ2-7	CASSLAGYEQYF	ORF1ab
HC2_CAGCGACTCTGCTGTC-1	TRBV5-1	TRBJ2-7	CASSSGTGSYEQYF	S(ORF2)
HC2_CATATTCCAGGGTACA-1	TRBV7-2	TRBJ2-3	CASSPGLAGTDTQYF	ORF1ab
HC2_CCATGTCAGGACTGGT-1	TRBV7-3	TRBJ2-2	CASSLAGTGELFF	ORF1ab
HC2_CGCTATCGTTCCCTTG-1	TRBV27	TRBJ2-2	CASSLTGELFF	N(ORF9)
HC2_CGGAGTCAGCGTGAGT-1	TRBV7-9	TRBJ2-7	CASSSTYEQYF	ORF1ab
HC2_CTGTGCTTCAGCTGGC-1	TRBV12-4	TRBJ2-7	CASSLGLYEQYF	ORF1ab
HC2_GTAGTCAAGACGACGT-1	TRBV11-2	TRBJ2-1	CASSLGTGGGNEQFF	S(ORF2)
HC2_GTGGGTCAGCTGCGAA-1	TRBV25-1	TRBJ2-1	CASSLLAGALNEQFF	ORF1ab
HC2_GTTACAGGTTAAAGAC-1	TRBV6-2	TRBJ1-1	CASSYGGAEAFF	ORF1ab
HC2_GTTCATTAGACGCACA-1	TRBV12-3	TRBJ2-7	CASSLQGYEQYF	ORF1ab
HC2_GTTCTCGAGGCTCATT-1	TRBV4-3	TRBJ1-1	CASSLGWNTEAFF	ORF6

HC2_TAAGTGCTCCTTAATC-1	TRBV3-1	TRBJ2-7	CASSTRSYEQYF	ORF1ab
HC2_TACTCATAGGCTAGCA-1	TRBV20-1	TRBJ1-2	CSALGVGYTF	ORF1ab
HC2_TCAGGTAAGTTCGCGC-1	TRBV27	TRBJ1-1	CASSLGANTEAFF	ORF6
HC2_TCGCGTTTCAATACCG-1	TRBV11-2	TRBJ2-7	CASSLAATYEQYF	ORF1ab
HC2_TGAGGGACACGGCTAC-1	TRBV5-1	TRBJ2-7	CASSLGGGYEQYF	ORF1ab
Yh002_acute_AAAGATGCAAATACAG-1	TRBV05-01	TRBJ02-07	CASSFSGGYEQYF	S (ORF2)
Yh002_acute_AACTCTTAGTCCAGGA-1	TRBV09	TRBJ02-05	CASSEGGNQETQYF	ORF1ab
Yh002_acute_AAGGTTCGTGAGGGTT-1	TRBV05-04	TRBJ02-02	CASSLGGNTGELFF	ORF1ab
Yh002_acute_ACGCCAGTCCGCGTTT-1	TRBV07-02	TRBJ02-07	CASSFGGNEQYF	ORF1ab
Yh002_acute_ACTTACTAGCTGTCTA-1	TRBV28	TRBJ02-07	CASSFSYEQYF	ORF1ab
Yh002_acute_ACTTGTTTCCTAGAAC-1	TRBV07-03	TRBJ02-01	CASSLISSSYNEQFF	ORF1ab
Yh002_acute_AGACGTTCAAGAGTCG-1	TRBV07-09	TRBJ02-03	CASSPDISTDTQYF	S (ORF2)
Yh002_acute_AGCATACAGTGGGCTA-1	TRBV07-09	TRBJ02-01	CASSPLDRGAYNEQFF	S (ORF2)
Yh002_acute_AGCGTCGGTCCAAGTT-1	TRBV07-09	TRBJ02-03	CASSPDISTDTQYF	S (ORF2)
Yh002_acute_AGGGATGAGCTGCCCA-1	TRBV04-01	TRBJ02-07	CASSQVWGSSYEQYF	S (ORF2)
Yh002_acute_ATCATCTTCTACTCAT-1	TRBV07-09	TRBJ02-07	CASSLGSAYEQYF	ORF1ab
Yh002_acute_ATCTACTTCCACGTTC-1	TRBV09	TRBJ02-02	CASSVGGTGELFF	ORF1ab
Yh002_acute_CAACTAGAGCGATATA-1	TRBV04-01	TRBJ02-03	CASSQSSGGTDTQYF	ORF1ab
Yh002_acute_CACATTTAGAGTAAGG-1	TRBV05-01	TRBJ01-01	CASSLGENTEAFF	ORF6
Yh002_acute_CACCACTGTAGCCTCG-1	TRBV19	TRBJ02-02	CASSVANTGELFF	S (ORF2)
Yh002_acute_CCTCAGTTCTTCATGT-1	TRBV07-02	TRBJ02-07	CASSLVSYEQYF	ORF1ab
Yh002_acute_CGAACATGTGGTCTCG-1	TRBV12-03	TRBJ02-07	CASSLDSYEQYF	ORF1ab
Yh002_acute_CGAATGTGTCTCGTTC-1	TRBV09	TRBJ01-06	CASSEAGDNSPLHF	ORF1ab
Yh002_acute_GAATAAGTCGTTACGA-1	TRBV11-01	TRBJ02-03	CASSSRDRIYTDTQYF	S (ORF2)
Yh002_acute_GACGTGCGTAGCCTAT-1	TRBV28	TRBJ02-07	CASSFGGGEQYF	ORF1ab
Yh002_acute_GCTGGGTAGAGCCTAG-1	TRBV03-01	TRBJ02-07	CASSQGLAGGYEQYF	ORF1ab
Yh002_acute_GGACATTAGTTACCCA-1	TRBV07-09	TRBJ02-03	CASSPDISTDTQYF	S (ORF2)
Yh002_acute_GGATGTTGTACCGCTG-1	TRBV13	TRBJ02-01	CASSWGLAGGINEQFF	S (ORF2)
Yh002_acute_GGCCGATCAATGGACG-1	TRBV05-01	TRBJ02-07	CASSLGQGSYEQYF	S (ORF2)
Yh002_acute_GGCGACTAGATAGCAT-1	TRBV09	TRBJ02-01	CASSVGGNEQFF	ORF1ab
Yh002_acute_GGGTCTGGTGGGTATG-1	TRBV07-09	TRBJ02-03	CASSGGTSGFTDTQYF	S (ORF2)
Yh002_acute_GGTGAAGGTAGCTGCC-1	TRBV02	TRBJ02-07	CASSDGTGSYEQYF	S (ORF2)
Yh002_acute_GGTGTTAGTCGGCATC-1	TRBV19	TRBJ02-07	CASSAPGAYEQYF	ORF1ab
Yh002_acute_GTCACAAAGCGCTTAT-1	TRBV05-01	TRBJ02-02	CASSSLNTGELFF	S (ORF2)
Yh002_acute_GTGCAGCTCTCGTTTA-1	TRBV11-03	TRBJ02-01	CASSLAGLSNEQFF	ORF1ab
Yh002_acute_GTTTCTACACCAGGCT-1	TRBV05-01	TRBJ02-02	CASSSLNTGELFF	S (ORF2)
Yh002_acute_TATTACCAGGTAGCTG-1	TRBV05-01	TRBJ01-01	CASSLGENTEAFF	ORF6
Yh002_acute_TCACGAATCGAACGGA-1	TRBV07-09	TRBJ02-03	CASSPDISTDTQYF	S (ORF2)
Yh002_acute_TCTTCGGAGTGTGAAT-1	TRBV07-09	TRBJ02-03	CASSPDISTDTQYF	S (ORF2)
Yh002_acute_TCTTCGGCAAGTCTGT-1	TRBV12-04	TRBJ01-03	CASSLGAGNTIYF	ORF1ab
Yh002_acute_TGGCCAGGTACTCGCG-1	TRBV13	TRBJ02-03	CASSLGSDTQYF	ORF1ab
Yh002_acute_TGGCGCAAGACACTAA-1	TRBV07-09	TRBJ02-03	CASSPDISTDTQYF	S (ORF2)
Yh002_acute_TTGGAACTCTGAAAGA-1	TRBV09	TRBJ02-03	CASSVVGRSTDTQYF	ORF1ab
Yh002_post_AAAGATGCATGTCCTC-1	TRBV07-09	TRBJ02-03	CASSPDISTDTQYF	S (ORF2)
Yh002_post_AACTCCCCAAGTACCT-1	TRBV09	TRBJ02-02	CASSVGGTGELFF	ORF1ab
Yh002_post_AGAGTGGTCTGATACG-1	TRBV14	TRBJ02-07	CASSQDRYEQYF	ORF1ab
Yh002_post_AGCAGCCCACTCGACG-1	TRBV07-09	TRBJ02-03	CASSPDISTDTQYF	S (ORF2)
Yh002_post_ATCCACCAGTGTCCAT-1	TRBV05-06	TRBJ02-07	CASSPSDLSYEQYF	S (ORF2)
Yh002_post_ATTGGACCAGCCAGAA-1	TRBV05-06	TRBJ02-07	CASSLGAYEQYF	ORF1ab
Yh002_post_CAAGAAAGTCAACTGT-1	TRBV02	TRBJ02-07	CASSAGTGAYEQYF	S (ORF2)

Yh002_post_CAAGTTGTCCCTCAGT-1	TRBV28	TRBJ02-03	CASSSLFRGGADTQYF	S (ORF2)
Yh002_post_CAGTCCTCATGATCCA-1	TRBV12-04	TRBJ02-05	CASSLAGGETQYF	ORF1ab
Yh002_post_CCATTCGGTTGGGACA-1	TRBV12-03	TRBJ02-07	CASTGGGYEQYF	ORF1ab
Yh002_post_CCCAGTTTCGCGGATC-1	TRBV05-04	TRBJ02-02	CASSLGGNTGELFF	ORF1ab
Yh002_post_CCTACACAGCTGGAAC-1	TRBV05-06	TRBJ02-05	CASSLGETQYF	ORF1ab
Yh002_post_CCTTCGAGTGGCCCTA-1	TRBV07-06	TRBJ02-01	CASSPGNEQFF	S (ORF2)
Yh002_post_CGAGAAGTCTTGAGAC-1	TRBV12-03	TRBJ02-03	CASSLTPESQYF	ORF1ab
Yh002_post_CGGACGTTCACCATAG-1	TRBV07-09	TRBJ02-05	CASSSETGQETQYF	ORF1ab
Yh002_post_CGTTCTGGTGTAACGG-1	TRBV07-09	TRBJ02-03	CASSPDISTDTQYF	S (ORF2)
Yh002_post_CTACATTGTTCGTGAT-1	TRBV09	TRBJ02-01	CASSLGQGEYNEQFF	N(ORF9)
Yh002_post_CTCAGAACACCGCTAG-1	TRBV07-02	TRBJ02-01	CASSLLAGGGNEQFF	ORF1ab
Yh002_post_GAAACTCCAAGTAGTA-1	TRBV27	TRBJ02-02	CASSLTGELFF	N(ORF9)
Yh002_post_GCATGCGTCGGCGGTT-1	TRBV05-06	TRBJ02-05	CASSLGETQYF	ORF1ab
Yh002_post_GCTGCTTTCACATGCA-1	TRBV11-02	TRBJ02-05	CASSLGTGETQYF	ORF1ab
Yh002_post_GCTGGGTTCCATTCTA-1	TRBV18	TRBJ01-01	CASSPGMNTEAFF	ORF6
Yh002_post_GTTCGGGTCCCAACGG-1	TRBV07-09	TRBJ02-03	CASSPDISTDTQYF	S (ORF2)
Yh002_post_TAGAGCTTCTTTAGGG-1	TRBV07-06	TRBJ02-02	CASSPTGTGELFF	ORF1ab
Yh002_post_TGACTTTGTCTTGTCC-1	TRBV06-05	TRBJ02-07	CASSYGGGYEQYF	ORF1ab
Yh002_post_TGGTTAGAGCTTTGGT-1	TRBV19	TRBJ01-01	CASSIGTGPNTEAFF	ORF1ab
Yh002_post_TTCTACACACAGACTT-1	TRBV12-04	TRBJ01-01	CASSSSDMNTEAFF	S (ORF2)
Yh004_acute_ACGCAGCTCAGTGCAT-1	TRBV03-01	TRBJ02-01	CASSQVLATYNEQFF	ORF1ab
Yh004_acute_AGAATAGCAGCTTAAC-1	TRBV18	TRBJ02-07	CASSLIGGEQYF	ORF1ab
Yh004_acute_CCTACACAGTTGAGAT-1	TRBV09	TRBJ01-01	CASSVGGNTEAFF	ORF1ab
Yh004_acute_CGAGAAGCACTTAAGC-1	TRBV07-06	TRBJ01-01	CASSWGMNTEAFF	ORF6
Yh004_acute_CGAGCCATCTACTTAC-1	TRBV05-06	TRBJ02-05	CASSLGETQYF	ORF1ab
Yh004_acute_CGCTATCAGACTGTAA-1	TRBV07-09	TRBJ02-03	CASSSGLAGTDTQYF	ORF1ab
Yh004_acute_CGGACTGGTCTGATCA-1	TRBV09	TRBJ01-05	CASSVGTNSNQPQHF	ORF1ab
Yh004_acute_CGTAGGCAGTCCGGTC-1	TRBV03-01	TRBJ02-01	CASSQVLATYNEQFF	ORF1ab
Yh004_acute_CTCGGAGTCCAAACAC-1	TRBV02	TRBJ01-01	CASSEGANTEAFF	ORF6
Yh004_acute_CTTCTCTAGTGCTGCC-1	TRBV04-01	TRBJ02-07	CASSQDHRGGYEQYF	ORF1ab
Yh004_acute_GAACATCGTATAGGGC-1	TRBV27	TRBJ01-01	CASSFGLNTEAFF	ORF6
Yh004_acute_GAGCAGACATTTCACT-1	TRBV28	TRBJ02-02	CASSDRNTGELFF	S(ORF2)
Yh004_acute_GAGGTGAAGGCTAGAC-1	TRBV27	TRBJ01-01	CASSFGLNTEAFF	ORF6
Yh004_acute_GCAAACTCACAGACTT-1	TRBV27	TRBJ01-01	CASSFGLNTEAFF	ORF6
Yh004_acute_GCGCCAAGTTTGGCGC-1	TRBV12-03	TRBJ02-01	CASSLGWYEQFF	ORF1ab
Yh004_acute_GGAAAGCGTGCAGTAG-1	TRBV28	TRBJ02-07	CASSLSGSYEQYF	ORF1ab
Yh004_acute_GGGATGACAAATTGCC-1	TRBV27	TRBJ01-01	CASSFGLNTEAFF	ORF6
Yh004_acute_GTACGTATCGTCCGTT-1	TRBV10-02	TRBJ02-07	CASRDPTTYEQYF	ORF1ab
Yh004_acute_GTCTTCGAGACAATAC-1	TRBV12-03	TRBJ02-07	CASSLGGSYEQYF	N(ORF9)
Yh004_acute_GTTACAGCATCGGTTA-1	TRBV03-01	TRBJ02-01	CASSQVLATYNEQFF	ORF1ab
Yh004_acute_TACGGATAGTTAACGA-1	TRBV09	TRBJ01-01	CASSVGGNTEAFF	ORF1ab
Yh004_post_AACCGCGGTACGCTGC-1	TRBV07-09	TRBJ02-07	CASSLETGSYEQYF	ORF1ab
Yh004_post_AACTCTTGTGCAGACA-1	TRBV29-01	TRBJ02-01	CSVLGLAGDSYNEQFF	S(ORF2)
Yh004_post_AAGGCAGGTCAACATC-1	TRBV05-01	TRBJ02-07	CASSFDSYEQYF	ORF1ab
Yh004_post_ACACCCTTCCGCGTTT-1	TRBV07-09	TRBJ02-02	CASSYTGELFF	S(ORF2)
Yh004_post_ACACCGGTCCGCAGTG-1	TRBV07-02	TRBJ02-07	CASSSSYEQYF	ORF1ab
Yh004_post_ACATACGGTAATCACC-1	TRBV09	TRBJ01-01	CASSVGGNTEAFF	ORF1ab
Yh004_post_ACTGTCCCATGCATGT-1	TRBV10-01	TRBJ02-01	CASTPGDSNEQFF	ORF7a
Yh004_post_AGGTCCGGTCAATGTC-1	TRBV24-01	TRBJ02-07	CATSDPDIYEQYF	S(ORF2)
Yh004_post_ATTCTACCAGTTCCCT-1	TRBV05-01	TRBJ01-04	CASSLGNEKLFF	ORF1ab

Yh004_post_CAGCGACTCCAGAGGA-1	TRBV18	TRBJ02-01	CASSPQDNEQFF	ORF1ab
Yh004_post_CAGCTGGTCTGCCAGG-1	TRBV05-06	TRBJ01-05	CASSLGSNQPQHF	ORF6
Yh004_post_CCAATCCGTCTACCTC-1	TRBV04-01	TRBJ02-07	CASSQVLAGAFEQYF	S(ORF2)
Yh004_post_CCCAGTTCATCGGGTC-1	TRBV07-03	TRBJ02-03	CASSPGLASTDTQYF	ORF1ab
Yh004_post_CCGTGGAGTTCCGGCA-1	TRBV04-01	TRBJ01-03	CASSLGQGSGNTIYF	S(ORF2)
Yh004_post_CCGTTCAGTACTCAAC-1	TRBV13	TRBJ02-01	CASSLGLGEQFF	ORF1ab
Yh004_post_CCTCTGAAGTGCCAGA-1	TRBV05-01	TRBJ01-02	CASSLGGGYTF	ORF1ab
Yh004_post_CGAGAAGTCAGCAACT-1	TRBV09	TRBJ01-01	CASSVGGNTEAFF	ORF1ab
Yh004_post_CGATTGAAGAGCCCAA-1	TRBV05-01	TRBJ02-02	CASSLGGELFF	ORF1ab
Yh004_post_CTAGCCTAGAAGGTGA-1	TRBV09	TRBJ02-03	CASSVGTGGLTDTQYF	ORF8
Yh004_post_CTCTGGTGTCGAGATG-1	TRBV11-02	TRBJ02-01	CASSLAGYNEQFF	ORF1ab
Yh004_post_CTGCGGAGTCAGATAA-1	TRBV04-02	TRBJ02-01	CASSQGGSYNEQFF	ORF1ab
Yh004_post_GAACCTACAATGTTGC-1	TRBV13	TRBJ02-02	CASSLNTGELFF	ORF1ab
Yh004_post_GATGAGGCAACACCTA-1	TRBV28	TRBJ02-01	CASSLGGYNEQFF	ORF1ab
Yh004_post_GCTCCTAAGTACGTTC-1	TRBV07-02	TRBJ01-01	CASSLGGTEAFF	ORF1ab
Yh004_post_GCTGCAGAGCCGATTT-1	TRBV05-06	TRBJ02-01	CASSPTGYNEQFF	S(ORF2)
Yh004_post_GTCACGGAGCCGCCTA-1	TRBV05-05	TRBJ02-07	CASSLGGTYEQYF	ORF1ab
Yh004_post_GTCGTAATCTTGCATT-1	TRBV07-09	TRBJ02-07	CASSFRDLPYEQYF	ORF1ab
Yh004_post_TATTACCGTTAAGACA-1	TRBV09	TRBJ02-07	CASSVSSGPYEQYF	S(ORF2)
Yh004_post_TCAGCTCAGGGATCTG-1	TRBV07-09	TRBJ01-06	CASSSGQGVSPLHF	S(ORF2)
Yh004_post_TGGCCAGCATCCTAGA-1	TRBV12-03	TRBJ02-07	CASSFTYEQYF	ORF1ab
Yh004_post_TTCGAAGAGAGAACAG-1	TRBV09	TRBJ01-01	CASSVGGNTEAFF	ORF1ab
Yh004_post_TTGGAACGTTCGTCTC-1	TRBV04-01	TRBJ02-03	CASSQDRELAGGTDTQYF	ORF1ab
Yh004_post_TTTACTGTCACATGCA-1	TRBV27	TRBJ02-07	CASSPGTGGLYEQYF	ORF1ab
Yh015_acute_AACCGCGTCTGAGGGA-1	TRBV12-03	TRBJ02-07	CASSPRGDEQYF	ORF1ab
Yh015_acute_CCAGCGATCCCTCTTT-1	TRBV06-04	TRBJ02-03	CASSPTSGSTDTQYF	ORF1ab
Yh015_acute_CCTTACGCAGGACGTA-1	TRBV12-03	TRBJ01-01	CASSLGGQTEAFF	N
Yh015_acute_CTCACACGTGCTCTTC-1	TRBV19	TRBJ02-01	CASSTAGLYNEQFF	ORF1ab
Yh015_acute_CTCGAAAAGAAGGTTT-1	TRBV07-03	TRBJ02-03	CASSLGLAGADTQYF	ORF1ab
Yh015_acute_CTGATAGAGTTAAGTG-1	TRBV13	TRBJ02-07	CASSFGQGAYEQYF	S
Yh015_acute_CTTACCGCACCACGTG-1	TRBV09	TRBJ02-02	CASSVDTGELFF	ORF1ab
Yh015_acute_GACGGCTGTCGCCATG-1	TRBV27	TRBJ02-02	CASSFGTGELFF	ORF1ab
Yh015_acute_GATCGTAAGTCAAGGC-1	TRBV05-04	TRBJ02-07	CASSLQGYEQYF	ORF1ab
Yh015_acute_GATGAGGCATCGGGTC-1	TRBV13	TRBJ02-07	CASSDHSSYEQYF	S
Yh015_acute_GCAGCCACAGAGCCAA-1	TRBV12-03	TRBJ02-07	CASSLSGGGEQYF	ORF1ab
Yh015_acute_GGTGAAGGTCAACTGT-1	TRBV12-03	TRBJ02-05	CASSLAGVETQYF	S
Yh015_acute_TTCTACATCCGTAGTA-1	TRBV04-03	TRBJ02-06	CASSQDSSGANVLTF	ORF1ab
Yh017_acute_AGCGTCGCATCCCATC-1	TRBV12-3	TRBJ2-3	CASSLALSQEYF	ORF1ab
Yh017_acute_CCTTCGATCCACTCCA-1	TRBV12-4	TRBJ2-3	CASSSWDRTSTDTQYF	S
Yh017_acute_CGATCGGCATGTCCTC-1	TRBV5-1	TRBJ1-1	CASSLAGTVNTEAFF	ORF1ab
Yh017_acute_CGCGGTATCAGCTCTC-1	TRBV18	TRBJ2-7	CASSPRDRVPEQYF	ORF1ab
Yh017_acute_CGCGTTTCAATGAAAC-1	TRBV9	TRBJ2-1	CASSVGLAGVVDEQFF	S
Yh017_acute_CTTTGCGAGCGTTTAC-1	TRBV5-1	TRBJ2-7	CASSLEAYEQYF	ORF1ab
Yh017_acute_GGACAAGTCGCCGTGA-1	TRBV4-1	TRBJ2-7	CASSQGEGTTYEQYF	ORF1ab
Yh017_acute_GGGAATGTCACATACG-1	TRBV12-4	TRBJ2-3	CASSSWDRTSTDTQYF	S
Yh017_acute_TAGGCATTCAATAAGG-1	TRBV12-4	TRBJ2-7	CASSLGGAYEQYF	ORF1ab
Yh017_acute_TATGCCCTCACATGCA-1	TRBV12-4	TRBJ2-3	CASSSWDRTSTDTQYF	S
Yh017_acute_TTCTACACACCTTGTC-1	TRBV7-6	TRBJ2-7	CASSFDRGYEQYF	N
Yh018_acute_AAACCTGAGATGCCAG-1	TRBV6-2	TRBJ2-7	CASSYGYEQYF	N
Yh018_acute_AAAGATGGTCAAAGCG-1	TRBV3-1	TRBJ2-7	CASSLGGYEQYF	ORF1ab

Yh018_acute_AAGACCTGTCGCATAT-1	TRBV28	TRBJ2-7	CASSWAYEQYF	ORF1ab
Yh018_acute_AAGTCTGTCTGACCTC-1	TRBV28	TRBJ2-1	CASSFGSVGNEQFF	M
Yh018_acute_ACACTGAGTAGTGAAT-1	TRBV7-9	TRBJ2-3	CASSFGTSGVTDTQYF	S
Yh018_acute_ACGGGTCTCTTAACCT-1	TRBV11-3	TRBJ1-1	CASSPGANTEAFF	ORF6
Yh018_acute_ACTTTCAAGAAACGAG-1	TRBV6-5	TRBJ1-1	CASSYSGNTEAFF	ORF1ab
Yh018_acute_AGCTCCTGTATCTGCA-1	TRBV7-6	TRBJ2-1	CASSAGTVSYNEQFF	ORF1ab
Yh018_acute_CATGACAAGCCAGTAG-1	TRBV6-6	TRBJ2-1	CASSGPGLAEEQFF	ORF1ab
Yh018_acute_CCAATCCCAAGAGTCG-1	TRBV7-9	TRBJ1-1	CASSLDRGTEAFF	N
Yh018_acute_CCGTGGAGTGATGATA-1	TRBV28	TRBJ2-7	CASSLGRSYEQYF	ORF1ab
Yh018_acute_CGAATGTAGGATGGTC-1	TRBV28	TRBJ2-7	CASSFSSYEQYF	ORF1ab
Yh018_acute_CGCTATCTCAGAAATG-1	TRBV10-1	TRBJ1-1	CASSESTGATEEAFF	ORF1ab
Yh018_acute_CGCTGGACACTGTCGG-1	TRBV4-1	TRBJ2-1	CASSQVPGYNEQFF	S
Yh018_acute_CGGAGCTCAAACCCAT-1	TRBV27	TRBJ1-5	CASSLAGSNQPQHF	ORF1ab
Yh018_acute_CGGAGTCAGCTGCAAG-1	TRBV28	TRBJ2-7	CASSFSSYEQYF	ORF1ab
Yh018_acute_CGTAGGCCAAGCCGCT-1	TRBV12-3	TRBJ2-7	CASSFPGAEQYF	ORF1ab
Yh018_acute_CGTAGGCCACGAGGTA-1	TRBV7-3	TRBJ2-2	CASSFAGELFF	S
Yh018_acute_CTACATTCACGAAACG-1	TRBV7-9	TRBJ1-1	CASSLDRGTEAFF	N
Yh018_acute_CTCGGAGCACTGCCAG-1	TRBV5-1	TRBJ2-7	CASSPGYEQYF	ORF1ab
Yh018_acute_CTCGTCAGTATCAGTC-1	TRBV5-5	TRBJ2-7	CASSSGYEQYF	ORF1ab
Yh018_acute_CTGATCCTCAACACTG-1	TRBV7-9	TRBJ2-5	CASSLAGGETQYF	ORF1ab
Yh018_acute_CTGCCTAAGCAAATCA-1	TRBV12-4	TRBJ2-7	CASSDRDRVSGTEQYF	S
Yh018_acute_CTGCTGTAGAAGGTGA-1	TRBV13	TRBJ2-5	CASSLGMETQYF	ORF1ab
Yh018_acute_CTGTGCTTCAAGGTAA-1	TRBV6-6	TRBJ2-1	CASSYPDLTNEQFF	S
Yh018_acute_CTTTGCGAGACTGTAA-1	TRBV24-1	TRBJ2-5	CATSDFDETQYF	S
Yh018_acute_GACTAACTCTACCTGC-1	TRBV15	TRBJ2-3	CATSRDVGTDTQYF	N
Yh018_acute_GGACAGAGTGTGACGA-1	TRBV7-9	TRBJ2-1	CASSPSSGSSYNEQFF	ORF1ab
Yh018_acute_GGGACCTGTCTTTCAT-1	TRBV5-5	TRBJ2-5	CASSPGTSSWETQYF	ORF1ab
Yh018_acute_GTCGGGTAGGTGCTTT-1	TRBV29-1	TRBJ2-7	CSAGTYEQYF	ORF3a
Yh018_acute_GTCTTCGGTCATCCCT-1	TRBV11-2	TRBJ2-7	CASSLGSYEQYF	ORF1ab
Yh018_acute_GTGCGGTTCTCCAGGG-1	TRBV27	TRBJ2-1	CASSLVGTSYNEQFF	ORF3a
Yh018_acute_TCATTTGTCGTTTATC-1	TRBV29-1	TRBJ2-1	CSVGSGDYNEQFF	ORF1ab
Yh018_acute_TCTTTCCTCCCATTAT-1	TRBV3-1	TRBJ2-3	CASSQVLATTDTQYF	S
Yh018_acute_TGCCAAATCAACGCTA-1	TRBV24-1	TRBJ2-1	CATSDSDRSSYNEQFF	S
Yh018_acute_TGCCCTACATCTGGTA-1	TRBV3-1	TRBJ2-7	CASSQTLGTAYEQYF	S
Yh018_acute_TGCCCTATCATTGCCC-1	TRBV9	TRBJ2-5	CASSVGRGQETQYF	ORF1ab
Yh018_acute_TGGCCAGCAAGCCGTC-1	TRBV4-1	TRBJ2-7	CASSQGPGTTYEQYF	ORF1ab
Yh018_acute_TTTATGCCAGTTTACG-1	TRBV5-1	TRBJ1-1	CASSLQGLNTEAFF	ORF3a
Yh034_acute_AAAGATGGTTAAAGTG-1	TRBV7-9	TRBJ2-7	CASSQSYEQYF	ORF1ab
Yh034_acute_CCTTACGCATCCGTGG-1	TRBV7-2	TRBJ2-5	CASSLSGGASETQYF	ORF1ab
Yh034_acute_CGGACACAGCTTTGGT-1	TRBV5-1	TRBJ2-7	CASSLGQGAYEQYF	S
Yh034_acute_CGTCTACTCCCTCTTT-1	TRBV27	TRBJ2-1	CASSFGGTNEQFF	ORF1ab
Yh034_acute_CTACACCTCACAATGC-1	TRBV6-1	TRBJ2-7	CASSEGDRGLYEQYF	ORF1ab
Yh034_acute_CTCGAAAAGTAGGTGC-1	TRBV9	TRBJ2-1	CASSVGGNEQFF	ORF1ab
Yh034_acute_GACTGCGAGTGTCCAT-1	TRBV7-8	TRBJ2-5	CASSLDGETQYF	S
Yh034_acute_GTCATTTCAGCGTTCG-1	TRBV02	TRBJ2-7	CASGGGYEQYF	ORF1ab
Yh034_acute_TGACAACTCTGAGGGA-1	TRBV11-2	TRBJ2-7	CASSLGQGSYEQYF	S
Yh034_acute_TTCTCAAAGAGTTGGC-1	TRBV7-9	TRBJ2-7	CASSVSYEQYF	ORF1ab
Yh034_acute_TTGGCAAGTACCAGTT-1	TRBV7-8	TRBJ2-5	CASSLDGETQYF	S

Table S3 DEGs between Yh002/Yh004 and Yh034

B cell

	Gene	p_val	avg_log2FC	p_val_adj
**1**	IFIT3	2.6182E-106	2.003477203	8.781E-102
**2**	MX2	1.82923E-75	1.781990762	6.13486E-71
**3**	XAF1	8.70609E-75	2.215004839	2.91985E-70
**4**	RPS27	1.36462E-72	1.552485169	4.57665E-68
**5**	RPL36A	1.92218E-68	2.053289331	6.44659E-64
**6**	RPL26	4.05761E-68	1.801416238	1.36084E-63
**7**	MT-ATP6	1.67497E-65	1.925730877	5.61751E-61
**8**	IFI44L	6.77449E-63	2.112072914	2.27203E-58
**9**	RPL41	2.25724E-60	1.099213226	7.57032E-56
**10**	RPS15A	4.56669E-60	1.208886773	1.53158E-55
**11**	MTRNR2L12	2.24752E-57	1.896810111	7.53772E-53
**12**	BTG1	2.39343E-54	1.768714579	8.02709E-50
**13**	MT-ND3	8.68879E-54	1.594709345	2.91405E-49
**14**	RPL37	1.09745E-51	1.130235544	3.68063E-47
**15**	TRIM22	6.8254E-49	1.543299644	2.2891E-44
**16**	RPL36	3.99429E-47	1.074738125	1.3396E-42
**17**	RPL39	1.17944E-46	1.028383632	3.9556E-42
**18**	RPS29	1.60711E-46	1.155365336	5.38993E-42
**19**	HBB	1.68828E-46	3.720079717	5.66217E-42
**20**	RPL37A	2.63195E-45	1.094676885	8.82705E-41

DC

	Gene	p_val	avg_log2FC	p_val_adj
**1**	PTGDS	1.0671E-15	4.435305751	3.57883E-11
**2**	NME8	4.48648E-15	0.698776445	1.50468E-10
**3**	ANKRD36	9.97808E-14	0.654473669	3.34645E-09
**4**	MAP1A	1.21291E-12	1.912329812	4.06786E-08
**5**	LINC00996	1.67906E-12	1.237958485	5.63122E-08
**6**	USP18	1.73665E-12	0.963334599	5.82439E-08
**7**	SMIM5	2.17393E-12	1.287791142	7.29092E-08
**8**	GPM6B	3.47831E-12	0.952729739	1.16656E-07
**9**	ISG20	6.61725E-12	1.827535145	2.21929E-07
**10**	BLNK	1.51492E-11	1.392373977	5.08074E-07
**11**	ZFAT	2.87259E-11	1.345103253	9.63408E-07
**12**	SLA2	3.4008E-11	1.04184285	1.14056E-06
**13**	HIST1H4C	3.45115E-11	2.389243456	1.15745E-06
**14**	SAMD9L	5.25321E-11	1.224942841	1.76182E-06
**15**	CYB561A3	5.53771E-11	1.829877775	1.85724E-06
**16**	KCTD19	7.56956E-11	0.639702994	2.53868E-06
**17**	SLC15A4	9.2605E-11	1.616407144	3.10579E-06
**18**	CLIC3	1.15227E-10	2.026317848	3.86448E-06
**19**	RPL26	1.38462E-10	1.546843576	4.64375E-06
**20**	CARD11	1.43083E-10	1.191747188	4.79872E-06

Classical monocyte

	Gene	p_val	avg_log2FC	p_val_adj
**1**	ISG15	0	2.22294846	0.00E+00
**2**	LY6E	0	1.485176843	0
**3**	IFITM1	0	3.560941241	0.00E+00
**4**	IFITM3	0	1.770158481	0.00E+00
**5**	IFITM2	1.4897E-305	1.656219399	4.996E-301
**6**	IFIT1	1.0032E-293	1.947679681	3.3646E-289
**7**	IFIT3	8.3257E-285	1.967157509	2.7923E-280
**8**	MX1	1.5701E-266	1.637117749	5.2658E-262
**9**	RPL28	9.0432E-265	0.680905132	3.03E-260

**10**	MT2A	2.7472E-257	1.930402403	9.2135E-253
**11**	TMSB10	7.4529E-257	0.714317199	2.4996E-252
**12**	XAF1	3.3172E-244	1.550751931	1.1125E-239
**13**	RPL36A	2.0609E-242	1.626809726	6.91E-238
**14**	MX2	2.9991E-241	1.601645679	1.0058E-236
**15**	S100A6	4.6634E-235	0.750593405	1.564E-230
**16**	RSAD2	2.5912E-233	1.62245676	8.6903E-229
**17**	HLA-A	1.2196E-226	0.869731106	4.0902E-222
**18**	IFI27	6.6632E-221	1.487184925	2.2347E-216
**19**	ISG20	4.4755E-220	1.664223856	1.501E-215
**20**	HLA-C	6.918E-216	0.814253394	2.3202E-211

Non cassical monocyte

	Gene	p_val	avg_log2FC	p_val_adj
**1**	IFIT1	1.68863E-14	3.407117766	5.66333E-10
**2**	IFIT3	7.05961E-11	2.623981625	2.36765E-06
**3**	ISG15	7.40266E-11	2.555009325	2.4827E-06
**4**	OAS1	1.49362E-10	2.15775099	5.00929E-06
**5**	MX2	2.02661E-10	1.948518838	6.79685E-06
**6**	RPL36A	3.53044E-10	1.894177527	1.18404E-05
**7**	IFIT2	3.67136E-10	2.44895876	1.2313E-05
**8**	OAS3	3.6893E-10	1.698513328	1.23732E-05
**9**	TNFSF10	1.14878E-09	1.928920191	3.85278E-05
**10**	OASL	1.29508E-09	1.879725088	4.34345E-05
**11**	MX1	1.55212E-09	1.971522922	5.2055E-05
**12**	IFITM1	2.11417E-09	2.068232603	7.09049E-05
**13**	IRF7	2.62413E-09	2.102314865	8.80082E-05
**14**	SERPINA1	4.39676E-09	1.283667306	0.000147458
**15**	GIMAP7	5.67112E-09	1.811084107	0.000190198
**16**	HLA-A	8.2943E-09	1.190678631	0.000278174
**17**	IFITM2	1.90061E-08	1.025397843	0.000637425
**18**	USP18	1.18647E-07	1.259851986	0.003979193
**19**	HERC5	1.23739E-07	1.352615349	0.004149949
**20**	XAF1	1.24898E-07	2.135162356	0.004188817

CD8+ effector & memory T cell

	Gene	p_val	avg_log2FC	p_val_adj
**1**	ISG15	4.19E-229	2.357992358	1.40E-224
**2**	MT-ATP6	8.51E-212	1.755952555	2.86E-207
**3**	IFI6	7.39E-205	1.888638799	2.48E-200
**4**	MTRNR2L12	3.14E-204	1.947737376	1.05E-199
**5**	XAF1	4.83E-182	1.78295921	1.62E-177
**6**	LY6E	3.61E-179	1.238654916	1.21E-174
**7**	HLA-C	2.34E-178	0.683600552	7.86E-174
**8**	RPL26	1.47E-162	1.180043222	4.94E-158
**9**	RPS27	3.59E-144	0.650695144	1.21E-139
**10**	RPL36A	4.28E-141	1.48749701	1.44E-136
**11**	MX1	1.46E-138	1.610132176	4.90E-134
**12**	IFI44L	3.50E-134	1.738397829	1.17E-129
**13**	IFITM1	1.75E-124	0.727986361	5.88E-120
**14**	RNF213	1.56E-120	1.237160484	5.23E-116
**15**	IRF7	6.33E-118	1.46819235	2.12E-113
**16**	HIST1H4C	8.66E-117	1.624509121	2.91E-112
**17**	KLRD1	3.12E-114	1.124111579	1.05E-109
**18**	MALAT1	1.42E-113	0.590100295	4.75E-109
**19**	ISG20	6.38E-112	1.167763154	2.14E-107
**20**	RPS15A	5.96E-109	0.64583327	2.00E-104

Table S4 DEGs between COVID-19 acute and recovered phase samples

NK cell

	Gene	p_val	avg_log2FC	p_val_adj
**1**	PTPRCAP	1.0449E-215	1.511527325	3.5043E-211
**2**	TXNIP	6.1203E-172	1.30530363	2.0526E-167
**3**	ARPC1B	1.1185E-148	1.251551887	3.7511E-144
**4**	FOS	4.811E-139	1.765679618	1.6135E-134
**5**	KLRD1	1.8323E-135	0.997544352	6.1452E-131
**6**	IFI6	3.5825E-124	1.576128543	1.2015E-119
**7**	LY6E	2.98993E-98	0.919791013	1.00276E-93
**8**	TSC22D3	6.73738E-96	0.962965681	2.25958E-91
**9**	IFITM1	5.07072E-76	0.55554878	1.70062E-71
**10**	EEF1G	1.00753E-68	1.002102321	3.37905E-64
**11**	PRF1	4.09593E-67	0.566232723	1.37369E-62
**12**	MYL12A	3.42817E-57	0.447101766	1.14974E-52
**13**	KLF6	7.84429E-56	0.924852091	2.63082E-51
**14**	HLA-C	9.16751E-52	0.257100586	3.0746E-47
**15**	JUN	9.41704E-45	1.01446555	3.15829E-40
**16**	CX3CR1	2.2084E-44	0.959573977	7.40655E-40
**17**	KLRF1	4.18151E-43	0.533389983	1.40239E-38
**18**	RHOC	6.5671E-43	0.84303398	2.20248E-38
**19**	XAF1	7.97493E-40	0.77074609	2.67463E-35
**20**	ECH1	9.43249E-40	0.717762712	3.16347E-35

Classical monocyte

	Gene	p_val	avg_log2FC	p_val_adj
**1**	IFI6	0	2.777264202	0.00E+00
**2**	S100A9	0	1.94183816	0
**3**	IFITM3	0	2.545493088	0.00E+00
**4**	IFI27	0	5.222949258	0.00E+00
**5**	ARPC1B	5.1104E-279	1.329771514	1.7139E-274
**6**	CLU	2.0115E-266	1.931052518	6.7462E-262
**7**	S100A8	1.5806E-264	1.542078253	5.3011E-260
**8**	CFL1	3.5163E-255	0.809023587	1.1793E-250
**9**	GAPDH	4.6635E-233	0.703191297	1.56E-228
**10**	ACTB	2.299E-231	0.546293826	7.7103E-227
**11**	PLAC8	7.3053E-231	1.815821384	2.4501E-226
**12**	B2M	6.5341E-227	0.752671143	2.1914E-222
**13**	JUN	1.481E-219	1.840464713	4.97E-215
**14**	RNASE2	9.0647E-217	1.978123331	3.0401E-212
**15**	LY6E	8.8167E-214	1.654427817	2.9569E-209
**16**	SH3BGRL3	4.092E-194	0.58484008	1.3724E-189
**17**	MNDA	1.171E-172	0.950636786	3.9273E-168
**18**	S100A12	1.4197E-167	1.43110352	4.7614E-163
**19**	MYL6	8.4238E-161	0.647265052	2.8252E-156
**20**	IFI30	2.1352E-155	1.007267103	7.1609E-151

Non cassical monocyte

	Gene	p_val	avg_log2FC	p_val_adj
**1**	IFI27	1.2299E-118	5.496485803	4.1248E-114
**2**	PLAC8	2.49098E-27	2.041243904	8.35426E-23
**3**	CD36	9.40957E-27	1.231570712	3.15578E-22
**4**	JUN	3.6021E-24	1.754376103	1.20807E-19
**5**	IFI6	4.06976E-24	2.208356103	1.36492E-19
**6**	ARPC1B	6.5912E-22	1.239686299	2.21056E-17
**7**	IFI30	1.04346E-21	1.702144047	3.49957E-17
**8**	CD63	3.30536E-20	1.783590327	1.10855E-15
**9**	FTL	4.02745E-20	0.798629092	1.35073E-15
**10**	CST3	8.82297E-20	0.898933739	2.95905E-15
**11**	TCN2	1.15782E-16	0.736349345	3.8831E-12
**12**	LGALS3BP	1.57828E-16	0.95203167	5.29323E-12
**13**	GABARAP	4.8881E-16	0.738205915	1.63937E-11
**14**	CD14	5.26636E-16	1.457746453	1.76623E-11
**15**	IFI44L	6.09303E-16	0.800159716	2.04348E-11
**16**	NCF1	6.85082E-16	1.857283207	2.29763E-11
**17**	IFITM1	8.90753E-16	2.316130807	2.98741E-11
**18**	S100A8	1.25876E-15	2.287376631	4.22164E-11
**19**	MS4A6A	1.36493E-15	1.360949216	4.57771E-11
**20**	IFITM3	3.07758E-15	1.132337102	1.03216E-10

CD8+ effector & memory T cell

	Gene	p_val	avg_log2FC	p_val_adj
**1**	PTPRCAP	6.17E-142	1.663298934	2.07E-137
**2**	FOS	3.62E-113	2.223110105	1.21E-108
**3**	TXNIP	1.55E-93	1.187827937	5.21E-89
**4**	B2M	3.14E-89	0.414350081	1.05E-84
**5**	ARPC1B	1.64E-85	1.276759421	5.51E-81
**6**	EEF1G	2.17E-56	1.218780754	7.27E-52
**7**	IFITM1	2.51E-56	0.660622218	8.41E-52
**8**	TSC22D3	6.60E-54	0.902063094	2.21E-49
**9**	DUSP1	1.88E-51	1.126439298	6.30E-47
**10**	HLA-E	9.43E-50	0.515549656	3.16E-45
**11**	MYL12A	1.20E-45	0.591886247	4.04E-41
**12**	IFI6	5.03E-44	1.242739366	1.69E-39
**13**	KLF6	8.61E-40	1.009037017	2.89E-35
**14**	LY6E	1.92E-33	0.726324871	6.44E-29
**15**	ACTB	7.47E-32	0.471611947	2.50E-27
**16**	HLA-C	8.52E-32	0.341328361	2.86E-27
**17**	CCND3	4.03E-29	0.690848676	1.35E-24
**18**	TMSB4X	3.23E-28	0.320320301	1.08E-23
**19**	CX3CR1	3.28E-28	1.079879207	1.10E-23
**20**	KLRD1	1.05E-27	1.045761792	3.54E-23
